# Ultra-low frequency magnetic energy focusing for highly effective wireless powering of deep-tissue implantable electronic devices

**DOI:** 10.1093/nsr/nwae062

**Published:** 2024-02-28

**Authors:** Yuanyuan Li, Zhipeng Chen, Yuxin Liu, Zijian Liu, Tong Wu, Yuanxi Zhang, Lelun Peng, Xinshuo Huang, Shuang Huang, Xudong Lin, Xi Xie, Lelun Jiang

**Affiliations:** Guangdong Provincial Key Laboratory of Sensor Technology and Biomedical Instrument, School of Biomedical Engineering, Shenzhen Campus of Sun Yat-Sen University, Shenzhen 518107, China; Guangdong Provincial Key Laboratory of Sensor Technology and Biomedical Instrument, School of Biomedical Engineering, Shenzhen Campus of Sun Yat-Sen University, Shenzhen 518107, China; School of Mechanical and Electrical Engineering, Guangzhou University, Guangzhou 510006, China; Guangdong Provincial Key Laboratory of Sensor Technology and Biomedical Instrument, School of Biomedical Engineering, Shenzhen Campus of Sun Yat-Sen University, Shenzhen 518107, China; Guangdong Provincial Key Laboratory of Sensor Technology and Biomedical Instrument, School of Biomedical Engineering, Shenzhen Campus of Sun Yat-Sen University, Shenzhen 518107, China; Guangdong Provincial Key Laboratory of Sensor Technology and Biomedical Instrument, School of Biomedical Engineering, Shenzhen Campus of Sun Yat-Sen University, Shenzhen 518107, China; Guangdong Provincial Key Laboratory of Sensor Technology and Biomedical Instrument, School of Biomedical Engineering, Shenzhen Campus of Sun Yat-Sen University, Shenzhen 518107, China; Guangdong Provincial Key Laboratory of Sensor Technology and Biomedical Instrument, School of Biomedical Engineering, Shenzhen Campus of Sun Yat-Sen University, Shenzhen 518107, China; State Key Laboratory of Optoelectronic Materials and Technologies, Guangdong Province Key Laboratory of Display Material and Technology, School of Electronics and Information Technology, Sun Yat-Sen University, Guangzhou 510006, China; State Key Laboratory of Optoelectronic Materials and Technologies, Guangdong Province Key Laboratory of Display Material and Technology, School of Electronics and Information Technology, Sun Yat-Sen University, Guangzhou 510006, China; Guangdong Provincial Key Laboratory of Sensor Technology and Biomedical Instrument, School of Biomedical Engineering, Shenzhen Campus of Sun Yat-Sen University, Shenzhen 518107, China; Guangdong Provincial Key Laboratory of Sensor Technology and Biomedical Instrument, School of Biomedical Engineering, Shenzhen Campus of Sun Yat-Sen University, Shenzhen 518107, China; State Key Laboratory of Optoelectronic Materials and Technologies, Guangdong Province Key Laboratory of Display Material and Technology, School of Electronics and Information Technology, Sun Yat-Sen University, Guangzhou 510006, China; Guangdong Provincial Key Laboratory of Sensor Technology and Biomedical Instrument, School of Biomedical Engineering, Shenzhen Campus of Sun Yat-Sen University, Shenzhen 518107, China

**Keywords:** ultra-low frequency, transcutaneous energy transmission, electromagnetic generator, implantable electronic device, deep tissue

## Abstract

The limited lifespan of batteries is a challenge in the application of implantable electronic devices. Existing wireless power technologies such as ultrasound, near-infrared light and magnetic fields cannot charge devices implanted in deep tissues, resulting in energy attenuation through tissues and thermal generation. Herein, an ultra-low frequency magnetic energy focusing (ULFMEF) methodology was developed for the highly effective wireless powering of deep-tissue implantable devices. A portable transmitter was used to output the low-frequency magnetic field (<50 Hz), which remotely drives the synchronous rotation of a magnetic core integrated within the pellet-like implantable device, generating an internal rotating magnetic field to induce wireless electricity on the coupled coils of the device. The ULFMEF can achieve energy transfer across thick tissues (up to 20 cm) with excellent transferred power (4–15 mW) and non-heat effects in tissues, which is remarkably superior to existing wireless powering technologies. The ULFMEF is demonstrated to wirelessly power implantable micro-LED devices for optogenetic neuromodulation, and wirelessly charged an implantable battery for programmable electrical stimulation on the sciatic nerve. It also bypassed thick and tough protective shells to power the implanted devices. The ULFMEF thus offers a highly advanced methodology for the generation of wireless powered biodevices.

## Teaser

A methodology of ultra-low frequency magnetic energy focusing is developed for wireless powering, which transmits energy through low-frequency electromagnetic waves with long-distance transmission and low energy attenuation through thick biological tissues.

## INTRODUCTION

Fully implantable electronic devices such as cardiac pacemakers [1,2], cardiovascular monitors [3,4] and deep-brain stimulators [5,6] have been widely developed in medical monitoring, diagnosis and treatments. For example, artificial cardiac pacemakers are effective treatments for bradycardia [7,8]. Such pacemakers generate electrical pulses to heart chambers to reduce symptoms, whereas implantable brain pacemakers use deep-brain electrical stimulation to treat Parkinson's disease [9,10], dystonia and other neuropsychiatric disorders. Implantable optogenetic devices [11,12] use light to activate specific brain activities in order to study brain circuits under natural conditions by releasing animals from tethered optical fibers. Existing implantable devices are based on power supply through replaceable batteries [13,14], self-powering supply [15,16] and wireless power transfer [17–19]. However, the key challenge for fully implantable electronic devices powered by disposable batteries is the limited battery life, which requires intermittent replacement via surgery, resulting in non-negligible health risks and medical costs. Self-powering supply involves the conversion of endogenous energy in the organism into electric energy through the circulatory, respiratory and digestive systems [20]. However, restricted organ movements, weak power and unstable energy collection present challenges. By contrast, wireless transcutaneous energy transfer techniques have been developed to transfer external energy to power implanted electronic devices across biological tissues. Currently, external energy sources, including ultrasound (through the piezoelectric [21] and triboelectric effect [22]), near-infrared light (through the photovoltaic effect) [23,24], heat (through the pyroelectric effect) [25] and magnetic field (through the electromagnetic effect) [26], have been used for wireless energy transfer. However, the acoustic, optical and thermal energies can be easily absorbed or reflected by the skin and tissue, resulting in thermal damage and severe energy attenuation through tissues. Therefore, these techniques generally suffer from issues of low energy transfer efficiency for thicker tissues.

**Figure 1.**
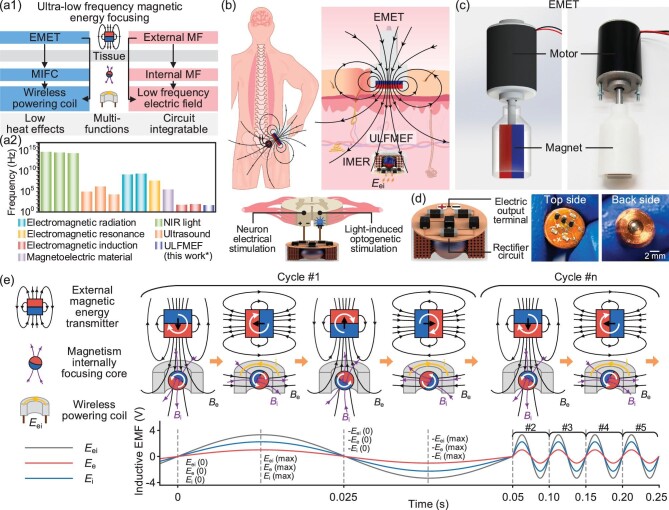
(a1) System design of the ULFMEF developed for wireless charging of implantable electronic devices. The ULFMEF system consists of an IMER and an EMET. (a2) Schematic showing the typical working frequency of ULFMEF, compared to other wireless energy transfer methods such as electromagnetic radiation, electromagnetic resonance, electromagnetic induction, magnetoelectric material, ultrasound and near-infrared light effects. (b) Illustration of the working mechanism of the ULFMEF system applied for wireless electrical stimulation and light-induced optogenetic stimulation in deep tissues. (c) Structure diagram and physical photograph of the EMET, which utilized a motor to control the rotation of a magnet to generate a low-frequency magnetic field. (d) Schematic diagram and photograph of the IMER combined with a rectifier circuit. (e) Schematic diagram showing the external/internal magnetism synergistic electricity-generating process in a typical cycle and during multiple cycles. The electricity is generated in the IMER through two pathways, including EMET-induced *E*_e_ via an external pathway and MIFC-induced *E*_i_ via an internal pathway, respectively.

Transcutaneous magnetic energy transfer uses oscillating electromagnetic fields as the energy transfer medium to wirelessly power implantable electronic devices, which is another promising technology to power implantable devices, owing to the advantages of high controllability, miniaturization and excellent biosafety. In this technology, coils or antennas are used to receive electromagnetic field energy for direct generation of electricity or for charging implantable batteries [27,28]. According to the electromagnetic effects, magnetic energy transfer generally includes electromagnetic radiation, magnetic resonance coupling and magnetic inductive coupling [29]. The electromagnetic radiation and magnetic resonance coupling are usually based on high field frequencies in the MHz-to-GHz frequency range (Fig. 1a2). However, high frequencies of the magnetic field of energy transfer are associated with a faster energy attenuation rate through the transmission distance, and thus have suffered from issues of limited tissue penetration depth. Magnetic energy transfer based on magnetic inductive coupling occurs when energy is coupled between transmitting and receiving coils through a magnetic field in the kHz-frequency range. Inductive coupling technology is highly sensitive to alignment and distance between coils, which is a challenge for efficient energy transfer. Low-frequency (<100 Hz) magnetic fields are not easily absorbed by biological tissues. However, low-frequency magnetic energy transfer has low power efficiency, requiring large-scale and high-performance magnetic field equipment to improve the power level. Therefore, wireless power supply for electronic devices implanted in deep tissues is highly challenging for existing technologies given the limited energy transmission distance. Moreover, because of the limited penetration capability, it is almost unfeasible for existing wireless transmission technologies to power implantable devices for humans wearing special metal suits such as protective suits or spacesuits, which are common in aerospace or military scenarios.

To the best of our knowledge, this study is the first to propose ultra-low frequency magnetic energy focusing (ULFMEF) for highly effective and robust wireless powering of deep-tissue implantable electronic devices. In the ULFMEF system, a portable magnetism transmitter is used to output a rotating magnetic field (5–50 Hz), which remotely drives the synchronous rotation of a miniature magnetism internally focusing core (MIFC) integrated within a pellet-like implantable device. This phenomenon generates an internal rotating magnetic field within the device that induces wireless electricity on the coupled metal coils next to the MIFC through magnetic inductive coupling. The ULFMEF transmits energy through low-frequency electromagnetic waves with long-distance transmission and low energy attenuation rate through biological tissues or most solid materials, which can effectively and safely produce wireless electrical output in deep tissues. The ULFMEF can achieve energy transfer across thick tissues (up to 20 cm) with excellent transferred power (4–15 mW). These performances are remarkably higher than those of existing wireless powering technologies, which overcomes the issue of electromagnetic energy absorption by tissue that occur for high (GHz–THz) and intermedium (kHz–MHz) ranges of frequency electromagnetic waves. We demonstrated that the ULFMEF integrated with the miniature circuit can wirelessly power implantable micro-LED devices to induce optical stimulation for optogenetics-activated behaviors of rats and wirelessly charge implantable rechargeable batteries to provide programmable electrical pulse stimulation on the sciatic nerve *in vivo*. Moreover, ULFMEF can wirelessly power implanted devices with superior penetration properties, avoiding the absorption of magnetic energy by thick and tough protective shells such as metal spacesuits, wood and stone materials. The effective, robust and versatile features of the ULFMEF render this methodology a highly promising wireless powering technology for a new generation of fully implantable biodevices.

## RESULTS AND DISCUSSION

The methodology of ULFMEF was developed for the wireless charging of implantable electronic devices based on the electromagnetic induction effect, as shown in Fig. 1. The magnetic energy transfer system mainly consists of an implantable magnetic energy receiver (IMER) and a portable external magnetic energy transmitter (EMET) (Fig. 1a1). The EMET generates an external magnetic field at ultra-low frequencies and the MIFC generates an internal magnetic field. The combined magnetic fields induce a low-frequency electric field in the wireless powering coil. Compared with other high-frequency technologies (Fig. 1a2), such as photovoltaic and electromagnetic effects, the ULFMEF system takes advantage of an ultra-low frequency magnetic field with low heat effects and multiple functions. The magnetic-core IMER was fabricated by encapsulating a NdFeB MIFC with high magnetization strength embedded inside a cylinder copper coil (Fig. 1b and Fig. S1), where additional circuits or stimulation electrodes were connected to the wireless powering coils. The magnetic flux density and magnetic field distribution of the MIFC were calculated and measured. The surface magnetic flux density of the magnetic core was measured as 144 mT (Fig. S2). The portable magnetic energy transmitter consists of a DC motor coupled to a speed controller to drive the rotation of a NdFeB magnet. The portable transmitter can generate a rotating magnetic field at ultra-low frequencies from 5 Hz to 50 Hz via the rotation of the motor-driving NdFeB magnet, where the rotating magnetic field penetrates the biological tissues and induces the electricity of the IMER through two pathways. In the external pathway, the rotating magnetic field of the transmitter can induce a magnetic flux change and generate an external magnetic-field-induced electric potential *E*_e_ in the coil. Also, importantly, in the internal pathway, the MIFC synchronously rotates with the external magnetic field due to the strong magnetic interaction between the driving magnet and MIFC. This results in the *in-situ* rotating magnetic field inside the coil and produces the internal magnetic-field-induced electric potential *E*_i_. The total generated electric potential *E*_ei_ in the IMER consists of the *E*_e_ and *E*_i_, where the existence of the MIFC in the IMER can remarkably enhance the total electricity output compared to conventional magnetic energy transfer. In contrast to other wireless energy transfer methods such as electromagnetic radiation, induction and resonance, this ULFMEF methodology significantly increases the transmission distance and slows down the attenuation rate during the energy transfer. Moreover, this low-frequency and long-distance magnetic energy transfer mode of ULFMEF can penetrate deeper biological tissues with minimal energy loss and thermal damage. The effective power supply of implantable microelectronic devices creates the potential to perform electrical stimulation or optical stimulation in deep tissues. Figure 1c displays a structure diagram and physical photograph of the fabricated magnetic energy transfer system, where the magnetic energy transmitter is portable and convenient for self-operation by patients in daily life. The engineering schematics, including front view, isometric view and bottom view, show the actual dimensions of the EMET (Fig. S3). The measured surface magnetic flux density of the driving magnet was 260 mT, and when the distance increased to 5 cm and 10 cm, the magnetic flux densities were ∼15 mT and 3 mT, respectively (Fig. S4). Figure 1d shows the top side and back side of the IMER with a size of Φ 8 × 4 mm^3^ and a weight of only ∼1 g. A miniature circuit was integrated on the top side to rectify the generated *E*_ei_-induced alternative current into direct current and boost the voltage. The outer surface of the IMER was uniformly sealed with a medical resin layer with a thickness of ∼200 μm to ensure biocompatibility (Figs S5 and S6).

The theory of the ULFMEF mechanism is analyzed in Fig. 1e. Due to the strong magnetic attraction (Fig. S7), the MIFC rotates according to the rotation of the driving magnet. Meanwhile, the rotation of the MIFC is hindered by the magnetic force generated by the induced current and the dynamic friction between the ball and coil (Fig. S8). The unavoidable friction between the magnetic ball and the coil generates heat loss. To minimize energy loss, the magnetic ball, when combined with the coil, is envisaged to be designed as a hybrid triboelectric-electromagnetic nanogenerator. This design aims to convert the inevitable friction into an alternative form of energy output [30–32]. The magnetic fields of both the driving magnet and MIFC exhibit a similar direction at the coil, resulting in superposition of the magnetic fields and effectively promoting the electromagnetic induction in IMER. The periodic fluctuations of the magnetic flux density caused by the rotation of the driving magnet and MIFC create two types of induced electric potentials *E*_e_ and *E*_i_ in the coil, respectively. Thus, based on Faraday's law of electromagnetic induction, the induced electromotive force *V*_emf_  *(equals to E*_ei_) in the IMER under the superposition of two magnetic fields can be derived as follows:


(1) \begin{eqnarray*}
{{E}_{{\mathrm{ei}}}} &=& {{V}_{{\mathrm{emf}}}} = - NS\frac{{d\Phi }}{{dt}} = - NS\frac{{d\left( {{{\Phi }_{\mathrm{e}}} + {{\Phi }_{\mathrm{i}}}} \right)}}{{dt}} \\
&=& - NS\frac{{d\left[ {\overline {{{B}_{\mathrm{e}}}} \cos\! \left( {\omega t + \delta } \right) + {{B}_{\mathrm{i}}}\cos\! \left( {\omega t} \right)} \right]}}{{dt}}\\
\end{eqnarray*}

where *N* and *S* present the turns and cross-sectional area of the coil, respectively, and *ω* is the rotation speed of the driving magnet. When the ULFMEF system reaches the dynamic equilibrium state, the driving magnet and the MIFC are in synchronous rotation with a phase difference *δ*. The phase difference *δ* is determined by the sum function of the magnetic force, coil resistance and friction force. The rotation angles of the driving magnet and MIFC are *φ* = *ωt* + *δ* and *θ* = *ωt*, respectively.

The magnetic flux density *B*_i_ of the MIFC, and the average magnetic flux density $\overline {{{B}_{\mathrm{e}}}} $ of the driving magnet at the IMER both synchronously induce electric potential in the IMER. When the IMER is positioned closer to the driving magnet, $\overline {{{B}_{\mathrm{e}}}} $ is much larger than *B*_i_ and the $\overline {{{B}_{\mathrm{e}}}} $-induced electricity *E*_e_ is larger than the *B*_i_-induced electricity *E*_i_. Nevertheless, if the IMER is far away from the driving magnet, *B*_i_ is much larger than $\overline {{{B}_{\mathrm{e}}}} $, and *E*_i_ is larger than *E*_e_, and thus the *E*_ei_ is mainly determined by *B*_i_. According to Equation (1), the induced current *I*(t) at a dynamic equilibrium state in IMER can be calculated as (see the detailed derivation in Fig. S8):


(2) \begin{eqnarray*}
I( t ) &=& - \frac{{NS}}{R}\sqrt {{{{\overline {{{B}_{\mathrm{e}}}} }}^2} + B_{\mathrm{i}}^2 + 2\overline {{{B}_{\mathrm{e}}}} {{B}_{\mathrm{i}}}{\rm cos}\delta } \cdot \omega\\
&& \cdot\, {\mathrm{cos}}\! \left[ {\omega t + \arctan\! \left( { - \frac{{{{B}_{\mathrm{i}}} + \overline {{{B}_{\mathrm{e}}}} {\mathrm{cos}}\delta }}{{\overline {{{B}_{\mathrm{e}}}} {\mathrm{sin}}\delta }}} \right)} \right]\\
\end{eqnarray*}

where *R* presents the internal resistance of the coil. Due to the periodic rotation of the driving magnet, the induced *E*_ei_ and *I*(t) also vary periodically, resulting in output fluctuations in each rotation cycle. For the uniform speed rotation of the driving magnet, the *E*_ei_ fluctuate in a sinusoidal profile, with the output voltage magnitudes varying between *E*_ei_ (max) and −*E*_ei_ (max).

**Figure 2.**
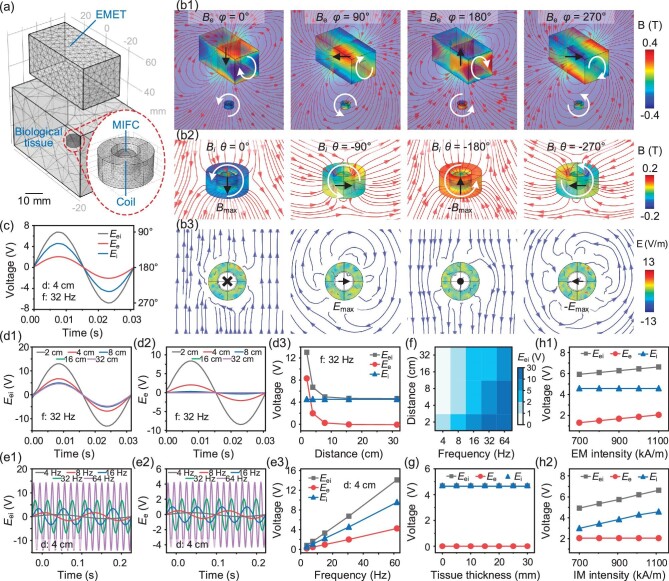
(a) Geometric model of the ULFMEF system in a COMSOL simulation, which consisted of an MIFC surrounded by a wireless powering coil implanted in biological tissue, with the EMET placed on top of the tissue. (b1) Dynamic magnetic field distribution of the ULFMEF system. The EMET rotated clockwise from 0° to 270°, and the MIFC synchronously rotated counterclockwise from 0° to −270°. (b2) Dynamic magnetic field lines around the IMER with the MIFC rotated from 0° to −270°, generating upward maximum magnetic flux density (*B*_max_) at 0° and downward maximum magnetic flux density (−*B*_max_) at −180°. (b3) Dynamic electric field lines around the IMER, generating parallel distribution at 0° and 180° and vortex distribution at 90° and 270°. (c) Induced electric potentials including *E*_ei_, *E*_e_ and *E*_i_ in a single cycle, calculated under a transmission distance of 4 cm with a magnetic field rotation frequency of 32 Hz. (d1–d3) Calculated *E*_ei_, *E*_e_ and *E*_i_ under different distances (2 cm, 4 cm, 8 cm, 16 cm and 32 cm) with a magnetic field frequency of 32 Hz. (e1–e3) Calculated *E*_ei_, *E*_e_ and *E*_i_ under a transmission distance of 4 cm with different frequencies (4 Hz, 8 Hz, 16 Hz, 32 Hz and 64 Hz). (f) Summary of calculated *E*_ei_ with different transmission distances and magnetic field rotation frequencies. (g) Calculated *E*_ei_, *E*_e_ and *E*_i_ inside biological tissue with different thicknesses (from 0 to 30 cm). (h1 and h2) Calculated *E*_ei_, *E*_e_ and *E*_i_ with different magnetization intensities (from 700 kA/m to 1100 kA/m) of external and internal magnetic fields.

Finite element analysis (FEA) was utilized to analyze the working mechanism of the ULFMEF system using FEA software COMSOL Multiphysics. The changes of magnetic and electric field distribution during the operation of the ULFMEF system, and the power generation performance of the system under different parameter conditions, were quantitatively calculated using the Electromagnetic Field module. The EMET was placed on top of a biological tissue, while the IMER (consisting of MIFC and wireless powering coil) was implanted inside the tissue. The geometric models of these objects and their relative positions were established according to the actual physical set-up (Fig. 2a and Fig. S9). The permeability and permittivity of these objects were set to 1.0 relative to air. The magnetic fields of the EMET and MIFC in the IMER, the magnetic strength of *B*_e_ and *B*_i_ were set to 700–1100 kA/m, and rotation frequency (frq) in the range of 1–64 Hz. The copper coil in the IMER was configured with a turn number of 3800 and a wire diameter of 0.06 mm, and the output voltage between the ending of coils was calculated. The magnetic field distribution of the system, as the driving magnet rotated, is shown in Fig. 2b1. As the magnetic field of the EMET rotated clockwise (from 0° to 180°), the MIFC was subjected to magnetic forces and generated a counterclockwise rotation, which induced a corresponding counterclockwise rotation of the magnetic field around the IMER. The magnetic field lines between the EMET and MIFC were converged and became dense at the MIFC, causing a stronger magnetic field near the IMER. Figure 2b2 and b3 show the magnetic field distribution and electric field variation around the IMER, respectively. When the rotation angle of the driving magnet was 0°, the majority of magnetic field lines of *B*_e_ and *B*_i_ were perpendicular to the cross-section of the IMER, resulting in the maximum magnetic flux density across the IMER. In this case, the electric potential in the IMER was zero and the electric field lines around the IMER exhibited a parallel distribution. As the driving magnet rotated to 90°, most magnetic field lines of *B*_e_ and *B*_i_ approached parallel alignment with the cross-section of the IMER. Consequently, the magnetic flux density across the IMER reached its minimum value, and the variation of magnetic flux density reached its maximum. The electric field surrounding the IMER appeared as a clockwise vortex, inducing a positive electric potential peak in the coil. As the driving magnet continued to rotate to 180°, the magnetic flux density across the IMER reached its inverse maximum. The electric field lines around the IMER changed to a parallel distribution and the induced electric potential in the coils returned to zero. When the driving magnet rotated to 270°, the magnetic field lines were reversed parallel to the IMER and the magnetic flux through the IMER reached a minimum once again. The electric field around the IMER showed a vortex distribution, resulting in a negative peak electric potential in the coil.

The induced electric potentials (including *E*_ei_, *E*_e_ and *E*_i_) in a single cycle were calculated under a distance (the thickness of biological tissue) of 4 cm between the EMET and IMER with a typical magnetic field rotation frequency of 32 Hz (Fig. 2c). The induced electric potential showed a sinusoidal curve variation with a period of ∼0.03 s (equivalent to 1/frq) and peak amplitudes of ∼6.7 V, 2 V and 4.6 V for *E*_ei_, *E*_e_ and *E*_i_, respectively (*E*_ei_ was the superposition of *E*_e_ and *E*_i_). For d = 4 cm, *E*_i_ occupied the dominant component of *E*_ei_, indicating that the induced electric potential in the IMER was mainly generated by the internal MIFC of the IMER. The *E*_ei_, *E*_e_ and *E*_i_ at different transmission distances were calculated to investigate how the electric performance of ULFMEF varied with distance (Fig. 2d1–d3). When the EMET and MIFC maintained the same rotating speed, the *E*_i_ maintained a stable peak voltage output of ∼4.6 V with increasing transmission distances (from 2 cm to 32 cm). In contrast, the peak values of *E*_e_ decayed rapidly (from 8.34 V to 0.0036 V), while the peak value of *E*_ei_ (defined as the output voltage) decreased more slowly (from 13 V to 4.71 V). Notably, in conventional magnetic energy transfer methods relying solely on external magnetic fields, the voltage generated by these systems typically rapidly decreases when the transmission distance significantly increases. While in the ULFMEF system, the output voltage can still produce a voltage >4.6 V even when the distance increases to 32 cm. This is attributed to the MIFC rotation, which provides an *in-situ* variable magnetic field that decays slowly with distance, allowing the entire system to maintain a high power-generation performance. In addition, the COMSOL model assumed that the EMET can generate enough magnetic force to drive the MIFC rotation at the same speeds. However, in actual experiments, the EMET failed to drive the MIFC rotation when the distance increased to a certain degree. The *E*_ei_, *E*_e_ and *E*_i_ at different magnetic field frequencies were further calculated to investigate the effect of frequency on the output performance of ULFMEF (Fig. 2e1–e3). Under a transmission distance of 4 cm, when the magnetic field rotation frequency increased (from 4 Hz to 64 Hz), the amplitudes of *E*_ei_, *E*_e_ and *E*_i_ showed a nearly linear increase. At frequencies up to 64 Hz, the peak value of *E*_ei_ was able to reach ∼14 V, while the peak value of *E*_i_ was close to ∼9.5 V. The output voltages of *E*_ei_ at different transmission distances and magnetic field frequencies were summarized in a heatmap plot (Fig. 2f). The results revealed that *E*_ei_ increased simultaneously with the increase of magnetic field frequencies (from left to right) and the decrease of transmission distance (from top to bottom), and *E*_ei_ exceeded 2 V for those frequencies >16 Hz. When the distance between the EMET and IMER was 32 cm, the dependence of ULFMEF output performance on the biological tissue thickness (from 0 to 30 cm) was evaluated (Fig. 2g). It was found that the thickness of biological tissue had limited effect on *E*_ei_, *E*_e_ and *E*_i_, mainly because the magnetization strength of the biological tissue was very close to that of air. Finally, the impact of magnetization intensity of the EMET and MIFC on the ULFMEF output performance was examined (Fig. 2h1 and h2), revealing that both the *E*_ei_ and *E*_e_ increased linearly with the external magnetization intensity, while *E*_ei_ and *E*_i_ increased linearly with the internal magnetization intensity.

**Figure 3.**
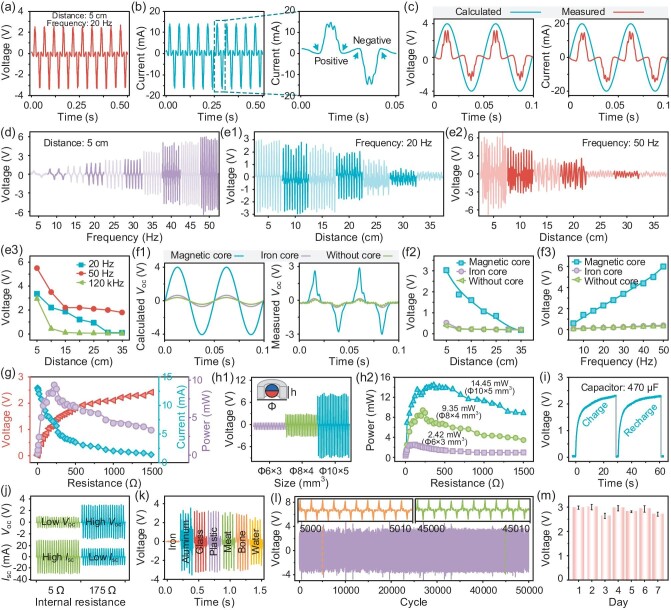
(a and b) Typical open-circuit voltage and short-circuit current waveforms of the IMER under a transmission distance of 5 cm and magnetic field frequency of 20 Hz. (c) Comparison of simulated results and experimentally measured results of the output voltage and current of the IMER. (d) Output voltage waveforms of the IMER under a transmission distance of 5 cm with different magnetic field rotation frequencies (from 5 Hz to 50 Hz). (e1–e3) Output voltage waveforms and statistical performance of the IMER at different transmission distances (from 5 cm to 35 cm) between the IMER and EMET under typical rotating frequencies of 20 Hz and 50 Hz. 120 kHz electromagnetic radiation was employed for comparison. (f1–f3) Calculation and experimental results of the output voltage waveforms and performance, to investigate the essential role of the MIFC in the IMER, where the IMER was designed without an MIFC or with an embedded iron core instead of an MIFC as control groups. (g) Measured output voltage, output current and output power of the IMER under different load resistances (from 1 Ω to 1500 Ω). (h1 and h2) The output voltage waveforms and output power of the IMER with sizes of Φ 6 × 3 mm^3^, Φ 8 × 4 mm^3^ and Φ 10 × 5 mm^3^, corresponding to different coil wire diameters (3 mm, 4 mm, 5 mm). (i) Charging and discharging capability of the IMER connected to a 470 μF capacitor in one minute. (j) Output voltage and current of the IMER with a coil wire diameter of 0.25 mm and 0.06 mm, with corresponding internal resistances of 5 Ω and 175 Ω. (k) Energy transfer capability of the ULFMEF across different material shells (iron, aluminum, glass, plastic, meat, bone and water) instead of air. (l) Electric output stability of the IMER over 50 000 cycles. (m) Electric output durability of the IMER over 7 days.

The electric output performance of the ULFMEF was experimentally measured (Fig. 3). The typical transmission distance was set to 5 cm from the driving magnet in the magnetization direction (Fig. S10) and the EMET rotation frequency was set to 20 Hz in experiments unless otherwise specified. Figure 3a and b present the typical output voltage and current waveforms in the presence of EMET rotation. The IMER generated a waveform with an open-circuit voltage (*V*_oc_) of 3 V and a short-circuit current (*I*_sc_) of 15 mA with an alternating profile corresponding to the EMET rotation frequency. 40 LEDs connected in parallel were illuminated by the IMER under a 20 Hz rotating magnetic field, as shown in Fig. S11. The output voltage and current waveforms were also compared to the COMSOL simulation results (Fig. 3c), where the results revealed that the experimental waveforms overlapped well with the calculated waveforms. The calculated electrical signals exhibited regular sinusoidal waveforms without any harmonic waves, while the experimental waveform displayed irregularities at the curve peaks and valleys likely attributed to the asynchronous vibration of the MIFC during rotation.

The electrical output performances of the IMER under different conditions, including transmission distances, magnetic field frequencies, external load resistances, IMER sizes, coil wire diameters and shielding materials, were further investigated. The magnetic field rotation frequency can be readily tuned by modifying the rotation speed or magnetic poles of the EMET (Fig. S12). The electrical output waveforms upon the increase of frequency is shown in Fig. 3d. The results found that increase of magnetic field rotation frequency significantly improved the electrical output *V*_oc_ and *I*_sc_ (Fig. S13a). The *V*_oc_ increased from 1.6 V to 6 V as the frequency increased from 5 Hz to 50 Hz, while the IMER can effectively output electricity of 1.6 V and 4 mA even at an ultra-low rotation frequency of 5 Hz. In theory, the larger the rotational frequency of the magnetic field, the higher the output voltage. To determine the optimal frequency, the rotational frequency of the magnetic field was increased from 10 Hz to 100 Hz and the electrical output signals of the IMER were measured at 15 cm and 20 cm from the EMET. According to the experimental results, the optimal frequency was 80 Hz at a transmission distance of 15 cm. Moreover, when the transmission distance increased to 20 cm, the optimal frequency decreased to 50 Hz. Therefore, 50 Hz was set as the maximum magnetic field frequency used in this paper (Fig. S14). When the electrical outputs were subjected to Fast Fourier Transform to obtain the frequency distribution (Fig. S15), the transformed characteristic peaks were well consistent with the magnetic field frequency, demonstrating the synchronous rotation behaviors of MIFC and EMET.

The electrical output waveforms (Fig. 3e1 and e2) and statistical performance (Fig. 3e3) of IMER at different separation distances between the IMER and EMET were then evaluated under typical rotation frequencies of 20 Hz and 50 Hz. The electric output from high-frequency electromagnetic radiation at 120 kHz was employed for comparison. The commercial wireless charging transmitter module, integrated into a XKT801 chip with a transmitting frequency of 120 kHz, was used as the high-frequency magnetic field transmitter. The transmitter coil had an outer diameter of 42 mm, an inner diameter of 21 mm, and a thickness of 2 mm. The Φ 8 × 4 mm^3^ IMER was used as the energy receiver. The receiver coil had an outer diameter of 8 mm, an inner diameter of 4.1 mm and a height of 4.1 mm. The wire diameter and internal resistance of the coil were 0.06 mm and 172.5–175.5 Ω, respectively. The diameter of the built-in magnetic ball was 4 mm. At 20 Hz ULFMEF operation, the electrical outputs of the IMER exhibited a slow attenuation from 2.78 V to 1.78 V upon increase of the transmission distance from 5 cm to 20 cm, and rapidly attenuated to 0.3 V when the transmission distance increased to 35 cm. This was mainly because the EMET can effectively drive the rotation of the MIFC to generate remarkable electrical outputs (>1.75 V) for a long distance of ∼20 cm, but the rotation of the MIFC became significantly compromised for distances exceeding 25 cm. At 50 Hz ULFMEF operation, the electrical outputs of the IMER showed a reduction from 5.42 V to 2.95 V when the distance increased from 5 cm to 10 cm. In contrast, the high-frequency electromagnetic radiation at 120 kHz showed a rapid attenuation from 2.95 V to 0.06 V when the distance exceeded 5 cm (Fig. S16), indicating the incapability of high-frequency electromagnetic radiation to transmit energy over longer distances. As the transmission distance further increased, the signal distortion and fluctuation of electrical outputs became more pronounced in time-domain plots. Additionally, unexpected characteristic peaks increased with transmission distance in frequency-domain plots (Fig. S17). This was most likely attributed to the step-missing, oscillating or crawling behaviors of the MIFC during rotation due to the gradual decrease in the magnetic interaction between the MIFC and EMET as the transmission distance increased.

The essential role of the MIFC in ULFMEF was also investigated through experiments and COMSOL simulation (Fig. 3f1–f3), where a bare copper coil without MIFC and a iron-core EMG were used as control groups. In these control groups, the energy transfer was dominated by the conventional pathway of electromagnetic induction via the external magnetic field. Both in experiments and COMSOL calculations, the IMER without MIFC or with an embedded iron core instead of MIFC both showed attenuated electrical waveforms with *V*_oc_ of ∼0.45 V, which is ∼7.5 times lower than the MIFC-IMER. Moreover, the electric outputs of the IMER were obviously improved ∼6-fold under a transmission distance of 15 cm (Fig. 3f2) and ∼15-fold under a magnetic field frequency of 50 Hz (Fig. 3f3) due to the existence of an MIFC compared to the control groups. This demonstrated that the MIFC assembled in the energy receiver can significantly enhance the power generation performance. The MIFC in the coil provided a non-decaying *in-situ* dynamic rotating magnetic field to induce electrical outputs in the IMER, thereby improving the electrical output performance, reducing field attenuation and enhancing transmission distance. The effects of external load resistance on the output voltage, output current and output power of the IMER were investigated (Fig. 3g). As the load resistances increased from 1 Ω to 1500 Ω, the output voltages also increased from 0.01 V to 2.4 V, while the output currents exhibited a decreasing tendency from 13 mA to 1.38 mA. Accordingly, the output power of the IMER increased at a lower resistance region (<200 Ω) and then decreased at a higher resistance region (>200 Ω), reaching a maximum output power *P*_max_ of ∼9.35 mW at a load resistance of ∼200 Ω.

To investigate the influence of the MIFC diameter on the output performance, the MIFC was designed with different diameters (3 mm, 4 mm, 5 mm), corresponding to three types of IMER sizes (Φ 6 × 3 mm^3^, Φ 8 × 4 mm^3^, Φ 5 × 10 mm^3^, Fig. 3h1 and h2). The results showed that the electrical output performance of the IMER dramatically increased with the sizes. In particular, the Φ 6 × 3 mm^3^ IMER produced *V*_oc_ of 1 V, *I*_sc_ of 10 mA and *P*_max_ of 2.42 mW; the Φ 8 × 4 mm^3^ IMER produced *V*_oc_ of 3 V, *I*_sc_ of 15 mA and *P*_max_ of 9.35 mW; and the Φ 10 × 5 mm^3^ IMER generated *V*_oc_ of 8 V, *I*_sc_ of 20 mA and *P*_max_ of 14.45 mW (Table S1). The charging and discharging capability of the IMER, connected to a typical capacitor of 470 μF, was investigated (Fig. 3i). The results demonstrated that the IMER possessed high energy charging capability (average charging rate of ∼36 μC/s), with the voltage of the capacitor boosted to 2.3 V within 30 s. The influence of the wire diameter and corresponding internal resistance of the wireless powering coil on the electrical performance of the IMER was also evaluated (Fig. 3j). By adjusting the wire diameters of the wireless powering coils (0.06 mm and 0.25 mm), the internal resistances corresponded to 175 Ω and 5 Ω, respectively, resulting in the IMER outputs of *V*_oc_ = 3 V (or *I*_sc_ = 15 mA) and *V*_oc_ = 1 V (or *I*_sc_ = 30 mA). Thus, the IMER can offer different power supply modes by adjusting the internal resistance. A higher internal resistance resulted in high voltage with low current, while a lower internal resistance produced low voltage with high current for output to an external circuit. The energy transfer capability of the ULFMEF across different material shells instead of air was evaluated. The IMER was placed in cubical boxes (50 × 50 × 50 mm^3^) made of different shielding materials including iron, aluminum, glass, plastic, meat, bone and water, and electrical output waveforms were measured (Fig. 3k and Fig. S13e). The output voltages were found to be in the order of ∼3 V for most non-conducting material shells, similar to the energy transfer through air, suggesting that the shielding effect of these materials can be ignored. However, when the IMER was shielded by an iron box, it only produced a weak output of *V*_oc_ = 20 mV and *I*_sc_ = 10 μA. This was due to the high magnetic permeability of iron, which weakened the magnetic energy transfer from the MIFC to IMER. This result suggested the potential of ULFMEF as an effective wireless power technology to bypass most shielding materials. However, it is unfeasible for existing wireless power technologies based on near-infrared light, ultrasound and high-frequency electromagnetic waves to power implantable devices for humans wearing special metal suits such as protective suits or spacesuits in aerospace or military scenarios. The stability of the electrical signals of the IMER was tested (Fig. 3l and m), and the results suggested that the IMER can stably output voltage waves with a peak of ∼3.2 V even under 50 000 cycles of rotation (Fig. 3l) and 500 000 cycles of rotation (Fig. S18a). The output stability test of IMER was also repeated for 7 days, with 50 000 cycles per day. The average *V*_oc_ over the 7-day test ranged from 2.7 V to 3 V with a fluctuation of <10% (Fig. 3m). To further investigate long-term stability, the voltage and current outputs of the IMER were measured over one month. The ULFMEF system consistently generated stable electrical signal outputs with voltage peaks fluctuating within a range of −3 V to 3 V (Fig. S18b), and current peaks varying within a range of −15 mA to 15 mA (Fig. S18c). After one month of operation, the IMER was disassembled into the internal magnetic ball and external copper coil. Optical images of the magnetic balls showed no damage to the surface (Fig. S18d). The magnetic hysteresis loops of the magnetic balls showed excellent magnetic properties after continuous rotation (Fig. S18e). The measured internal resistances showed no significant difference between experimental group and control group (Fig. S18f). The results indicated that the ULFMEF system exhibited long-term stability in its electrical performance and physical structure. The IMER possessed good stability and durability for long-term energy harvesting from the EMET.

**Figure 4.**
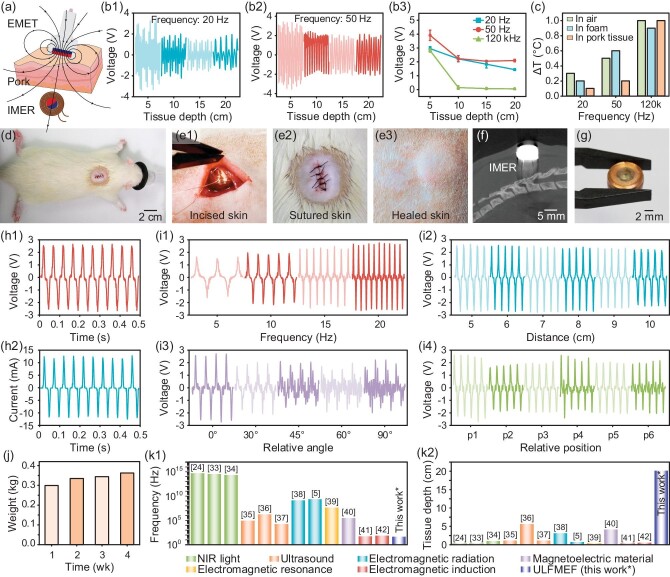
(a) Illustration of the ULFMEF system as an effective wireless power source for an implantable device under pork tissue. (b1–b3) electrical output waveforms and performance of the IMER implanted under pork tissue at different depths (from 5 cm to 20 cm), under typical rotation frequencies of 20 Hz and 50 Hz, with 120 kHz employed for comparison. (c) Temperature changes of the IMER placed in air, foam and pork tissue after working for 30 minutes under 20 Hz and 50 Hz, with 120 kHz electromagnetic radiation employed for comparison. (d) Photograph showing the IMER implanted under the skin on the back of a rat. (e1–e3) Photographs of the incised skin, sutured skin and healed skin (after 1 month) of the experimental rat before and after the IMER implantation. (f) Micro-CT image of the IMER implanted underneath the skin on the back of the rat for 1 month. (g) The implanted IMER retrieved from the tissue of the experimental rat after being implanted for 1 month. (h1 and h2) Typical open-circuit voltage and short-circuit current waveforms of the IMER implanted under the rat’s skin with a transmission distance of 5 cm and magnetic field frequency of 20 Hz. (i1–i4) Effects of magnetic field frequencies, transmission distances, relative angles and relative positions between the EMET and the IMER on the *in vivo* electrical output performance. (j) Recorded body weight of the rat implanted with the IMER over 4 weeks. (k1 and k2) Working frequency and tissue depth of the ULFMEF, compared with other wireless energy transfer technologies. The numbers of reference articles are indicated in brackets.

To utilize the ULFMEF as an effective wireless power source for bio-implantable devices, the electrical output performance was evaluated by implanting the IMER underneath thick tissue (using pork as a study model, Fig. 4a). The EMET was placed close to the porcine tissue (with thickness ranging from 5 cm to 20 cm) to power the IMER which was placed underneath the porcine tissue. The electrical output waveforms (Fig. 4b1 and b2) and statistical performance (Fig. 4b3) of the IMER buried in different tissue depths were measured under typical rotation frequencies of 20 Hz and 50 Hz, and the electrical outputs from high-frequency electromagnetic radiation at 120 kHz were employed for comparison. At 20 Hz ULFMEF operation, the IMER can generate *V*_oc_ of 3 V when buried underneath a 5-cm-thick porcine tissue. As the porcine tissue depth increased from 5 cm to 20 cm, the electrical outputs of the IMER slightly decreased from 3.12 V to 1.46 V (Fig. 4b1). At 50 Hz ULFMEF operation, the electrical outputs generated by the IMER displayed a reduction from 3.17 V to 1.86 V as the tissue thickness increased from 5 cm to 20 cm (Fig. 4b2). The electrical output performance of the IMER placed underneath tissue was not significantly lower than when the IMER was placed in air, suggesting the biological tissue did not compromise the magnetic energy transfer. In contrast, the electrical performance of the IMER under high-frequency electromagnetic radiation at 120 kHz showed a rapid attenuation from 2.9 V to 0.13 V when the porcine tissue depth exceeded 5 cm, indicating the inability of high-frequency electromagnetic radiation to transmit energy to deeper tissues.

The heat generated by the working IMER during ULFMEF operation was investigated. The IMER was placed in air, in foam and in pork tissue, respectively. The temperature changes of the IMER were measured after the ULFMEF system had been operating for 30 minutes under 20 Hz and 50 Hz magnetic fields. As a control, tissue temperature changes under high-frequency electromagnetic radiation at 120 kHz were employed for comparison (Fig. 4c). When the IMER was placed in air, the temperature increased 0.3°C, 0.5°C and 1.0°C after 30 minutes of operation under 20 Hz, 50 Hz and 120 kHz magnetic fields, respectively. When the IMER was placed in an adiabatic foam, the temperature increased 0.2°C, 0.6°C and 0.9°C after 30 minutes under 20 Hz, 50 Hz and 120 kHz magnetic fields, respectively. When the IMER was placed in pork tissue, the temperature changed 0.1°C, 0.2°C and 1.0°C after 30 minutes under 20 Hz, 50 Hz and 120 kHz magnetic fields, respectively (Fig. S19). Based on the experimental results, it can be seen that the IMER produced limited heat when operated in air, foam and pork tissue under a 20 Hz magnetic field, while a large temperature increase was observed under a 120 kHz magnetic field. The results suggested that the ULFMEF did not significantly increase the tissue temperature during operation, which was beneficial for biological safety without causing thermal damage to tissue.

Then the IMER was further tested *in vivo* by implantation in a living rat (Fig. 4d). The IMER was surgically implanted in the rat's back and was followed by skin suturing (Fig. 4e1 and e2), and the wound was well healed after one month (Fig. 4e3). The micro-CT (Inveon, Siemens, German) confirmed the stable implantation of the IMER beneath the rat's skin (Fig. 4f), and the implanted IMER can be retrieved from the tissue (Fig. 4g). The *in-vivo* electrical output performances of the ULFMEF were experimentally measured (Fig. 4h and i), with the typical transmission distance and the EMET rotation frequency set to 5 cm and 20 Hz. Figure 4h1 and h2 present the typical output voltage and current waveforms in the presence of EMET rotation. The IMER generated waveforms with an open-circuit voltage (*V*_oc_) of 2.8 V and a short-circuit current (*I*_sc_) of 13 mA with alternating profile, similar to those where the IMER and EMET were separated by an air environment. Moreover, the effects of magnetic field frequencies, transmission distances, relative angles and relative positions between the EMET and the IMER on the *in-vivo* electric output performance were further investigated (Fig. 4i). The results showed that increasing magnetic field frequencies from 5 Hz to 20 Hz significantly improved the electric output *V*_oc_ from 1.60 V to 2.64 V (Fig. 4i1). At 20 Hz magnetic field (Fig. 4i2), the electrical outputs exhibited slow attenuation from 2.6 V to 2.26 V upon increase of the transmission distances from 5 cm to 10 cm. The electrical output waveforms of the IMER upon different relative angles between the EMET and IMER were also measured (Fig. 4i3 and Fig. S20a). The output voltages of the IMER fluctuated in the range of 1.71 V to 2.66 V as the relative angles varied from 0° to 90° between the EMET and the rat dorsal surface. Additionally, the EMET was placed in six different locations (indicated as p1 to p6, as illustrated in Fig. S20b) relative to the IMER in the rat dorsal. The output voltages of the IMER were observed to fluctuate in the range of 1.79 V to 2.5 V at different locations. The above results indicated that the implanted IMER can stably output a considerable electrical power *in vivo*, and the presence of biological tissues did not obviously impair the wireless electrical output. The body weight of the rat implanted with an IMER was monitored over 4 weeks, and the results showed that the rat maintained a constant increase in weight from 300 g to 366 g (Fig. 4j) without noticeable behavioral abnormalities, indicating the biosafety of the implantation of the IMER.

The working frequency and tissue penetration depth of representative wireless transcutaneous energy transfer techniques, including near-infrared light, ultrasound, magnetic fields (high, medium and low frequencies) and magnetoelectric material, that are needed in order to generate sufficient power output (generally 1–10 mW) to power implantable devices, are summarized in Fig. 4k. The advantages and limitations of these techniques are compared to the ULFMEF in this work in Table S2. The near-infrared light and ultrasound can be easily absorbed or reflected by biological tissues with limited penetration depth, and cause thermal damage on tissue. The near-infrared light energy transfer relied on the photovoltaic effect of the optical device implanted in the subcutaneous site, based on near-infrared light ranges from 0.3 THz to 400 THz with high energy density. In representative works, near-infrared light energy is found to only power photovoltaic devices implanted in tissue with limited thickness. For example, Lyu *et al.* reported a photo-thermal-electric converter implanted in the abdominal cavity of a rabbit at a depth of 8.5 mm [33]. Jeong *et al.* designed an optically powered and controlled wireless optogenetic system using near-infrared light, which was implanted underneath a mouse's skin at a depth of 0.55 mm [34]. Ultrasound energy transfer converted external ultrasonic waves into electrical power through piezoelectric or triboelectric implanted devices [35], with the working frequency ranging from 20 kHz to 3 MHz, and tissue penetration depth up to ∼6 cm. For example, Piech *et al*. designed a wireless and implantable neural stimulator based on a piezoelectric transducer that can achieve electrical stimulation and recharge at a depth of 55 mm through *ex-vivo* porcine tissue [36]. Hinchet *et al*. demonstrated an ultrasound-vibrating triboelectric generator implanted underneath the skin at a depth of 10 mm [37]. Transcutaneous magnetic energy transfer utilizes oscillating electromagnetic fields as the medium to propagate through the tissue for wireless powering. The transmission efficiency of this method is affected by the magnetic field frequency. According to the frequency, magnetic energy transfer generally includes forms of electromagnetic radiation (high-frequency magnetic field), magnetic resonance coupling (middle-frequency magnetic field) and magnetic inductive coupling (low-frequency magnetic field) [20]. However, energy transfer through electromagnetic radiation and resonance is associated with faster energy attenuation rate within biological tissue. Thus, these methods have been suffering from issues of limited electricity output and limited tissue penetration depth (<5 cm). Zaeimbashi *et al*. reported an ultra-compact magnetoelectric antenna implanted underneath *in-vitro* mice brain tissue with a thickness of 6 mm, which can perform wireless energy harvesting under a high-frequency (2.51 GHz) electromagnetic field [5]. Bansal *et al*. reported a miniaturized implantable device wirelessly powered by a radio-frequency (1.5 GHz) system, which can deliver energy through 3-cm-thick porcine tissue [38]. For electromagnetic resonance, Sun *et al*. implanted a micro-LED in a mouse abdomen adjacent to the bladder with an implantation depth of 1.9 mm, which was wirelessly powered by a 50 MHz resonant magnetic field [39]. Similar to coupling coils, magnetoelectric materials can also convert magnetic fields into electric fields through mechanical coupling between magnetostrictive and piezoelectric layers. Chen *et al*. reported a magnetoelectric endovascular wireless and battery-free millimetric stimulator implanted in an *ex-vivo* model of porcine tissue at a depth of 4 cm, which was powered by a 345 kHz resonant magnetic field [40]. Low-frequency magnetic fields (<200 Hz) are not easily absorbed by biological tissues, but also possess high energy attenuation rate across transmission distances, and the energy density of low-frequency magnetic fields is relatively low. Therefore, low-frequency magnetic fields require the use of high-strength magnetic fields to generate sufficient energy to power implantable devices, yet the reported transmission depth is generally limited to 4 cm when utilizing a portable magnet set-up. For example, Lee *et al*. presented a magneto-mechano-triboelectric nanogenerator implanted underneath a mouse's skull (1 mm depth), which was powered by a 60 Hz stray magnetic field [41]. As an additional power supply option to temporary implants, a type of wireless magnetic energy harvester was composed of a rotating magnet serving as the transmitter and a flat antenna as the remote receiver. Its key advantages are its capability for operating at low frequencies and across a diverse range of scenarios. For example, Guo *et al.* introduced a bioresorbable system for wireless power transfer, serving as a stimulator for the sciatic nerve in a rat model at a magnetic field frequency and working distance of 83 Hz and 4 mm [42]. In this work, the ULFMEF is based on ultra-low frequency (5–50 Hz) magnetic energy, which is minimally absorbed by biological tissue as well as most non-conductive materials. Unlike the conventional energy transfer by low-frequency magnetic fields, the ULFMEF utilizes the magnetic field to drive the synchronous rotation of a magnetic core integrated within the implantable device. Because the magnetic core was placed close to the coupled coils of the device, the issue of rapid energy attenuation across the transmission distance of a low-frequency magnetic field can be better addressed. The ULFMEF had the potential to provide highly efficient and robust wireless power for electronic devices implanted in deep tissues up to 20 cm, while generating a reasonable electrical power output of 4–15 mW. The tissue penetration depth is remarkably higher than other existing wireless powering technologies. The biological safety of ULFMEF is also superior to other transcutaneous energy transfer techniques, as the ultra-low frequency magnetic energy rarely produces a thermal effect on tissue due to limited energy absorption by biological tissues.

**Figure 5.**
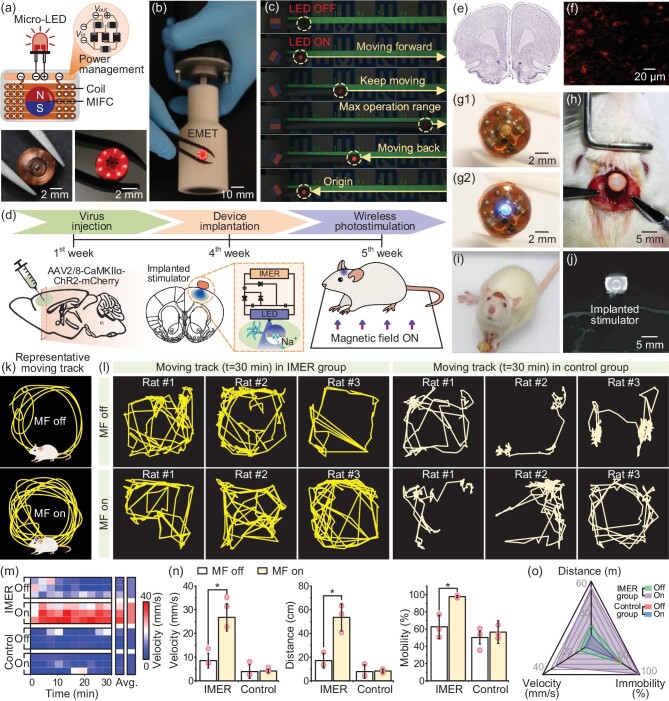
(a) Diagram illustration of the implanted micro-LED device for optogenetic neuromodulation, which can be wirelessly activated to emit light under the rotating magnetic field. (b) Demonstration of an IMER device integrated with eight micro-LEDs to be lighted by the EMET. (c) Demonstration of the ULFMEF system that realized long-distance energy transfer. The micro-LED device can maintain brightness up to a transmission distance of 35 cm. (d) Experimental procedure of micro-LED integrated IMER implantation for optogenetics-activated behavior studies of rats, including virus injection in the first week, micro-LED device implantation in the fourth week and wireless optogenetic neuromodulation in the fifth week. (e) Histological analysis of brain tissue after optogenetic virus injection to check for brain structure damage and inflammatory response. (f) Fluorescent signals of photosensitive ChR2 protein at M1, demonstrating its stable and full expression. (g1 and g2) Photograph of the micro-LED integrated IMER combined with an optical fiber. (h) Surgery implantation of the optoelectronic stimulator on the rat's skull. (i) Photograph of the rat implanted with the optoelectronic stimulator in the fifth week, keeping healthy without significant activity disorders. (j) Micro-CT image of the rat's head implanted with the micro-LED integrated IMER. (k) Illustration of the typical moving track profile of rats with or without stimulation by the micro-LED integrated IMER device. The micro-LED induced optoelectronic stimulation would activate the locomotion behaviors of rats. (l) Recorded moving tracks of the rats in the IMER group and control group (without optogenetic device implantation) under MF-on and MF-off states. (m) Heatmap plot summarizing instantaneous movement velocities within 30 minutes, of the rats in different groups. (n) Quantitative analysis of the average movement velocity, locomotion distance and mobility time duration of the rats in different groups. (o) Radar plot summarizing the locomotion distance, mobility time duration and velocity of the rats in the four testing groups.

The ULFMEF methodology was demonstrated to wirelessly power an implantable micro-LED device in the brain to generate optical stimulation for optogenetic neuromodulation. The micro-LED device can be wirelessly activated to emit light under a rotating magnetic field. The IMER was integrated with a power management circuit (Fig. 5a) to rectify the output current into direct current to power the micro-LEDs assembled on the IMER. For demonstration, an IMER device was integrated with eight micro-LEDs, and the micro-LEDs were observed to light up when a portable EMET (B = 260 mT) transmitted magnetic energy to them (Fig. 5b). To demonstrate that the ULFMEF system can realize long-distance energy transfer at an ultra-low field frequency, the micro-LED integrated IMER was moved forward and backward at a constant speed while being actuated by the EMET. The luminance of the micro-LEDs can maintain brightness until the transmission distance reached 35 cm (Fig. 5c and Video S1). Optical stimulation [43–45] is a powerful technique that allows specific control of neuronal activity spatiotemporally, by targeted expression and activation of light-sensitive proteins for regulating neural circuits and therapeutic interventions. A wireless and battery-free optoelectronic stimulator based on the IMER was developed (Fig. S21). The stimulator consisted of the IMER, the power management circuit and the μ-LED (wavelength: 460 nm) integrated with an optical fiber (diameter: 850 μm, and length: 1.5 mm) for transmitting light to the primary motor cortex. The micro-LED integrated IMER was implanted above the rat's skull in order to study the optogenetics-activated behaviors. The preliminary experiment proved that bone tissue will not absorb a low-frequency magnetic field, which may not induce attenuation in the electrical performance of the ULFMEF system (Fig. S22). The timeline for the whole experimental procedure was illustrated in Fig. 5d, where the experimental rats were injected with the photogenic virus AAV-CaMKIIa-ChR2-mCherry in the primary motor cortical area (M1) in the first week. The photosensitized protein was fully expressed in neurons 3 weeks after the injection. The histological analysis of brain tissue following optogenetic virus injection revealed an intact structure with no signs of damage or inflammatory response (Fig. 5e). Fluorescent signals from the photosensitive ChR2 protein were clearly observed at the target site (M1), demonstrating the stable and full expression of ChR2 protein (Fig. 5f). In the fourth week, the optoelectronic stimulator (Fig. 5g, diameter: 6 mm, and height: 4 mm) was implanted into the rat's head and the optical fiber was fixed through a drilled hole in the skull to reach the primary motor cortex (Fig. 5h). For optogenetic activation of the ChR2 protein, the micro-LED was lit up via the IMER driven by the EMET. The luminous intensities of the IMER-driven LED were investigated at different magnetic field frequencies from 10 Hz to 50 Hz and different transmission distances from 5 cm to 20 cm, as shown in Fig. S23. The IMER-driven LED can provide enough luminous power at a distance of several centimeters, which is sufficient for optogenetic stimulation. In the fifth week, the rats were observed to recover well and keep healthy without any significant activity disorders (Fig. 5i). Additionally, the micro-LED integrated IMER can properly work without breakage or position shifting after implantation (Fig. 5j) based on the micro-CT image. Upon activation of the EMET rotation, the micro-LED integrated IMER can emit blue light to excite the target neuron and activate the ChR2. For ChR2, when irradiated by a blue laser in the wavelength range of 400 nm to 480 nm, the covalently bound to ChR2 absorbs the photons, prompting a change in the molecular conformation of ChR2. Cation channels appear in the protein and the inward flows of Na^+^ and Ca^+^ ions then cause the rising intracellular potential, activating motor neurons and promoting the rats’ locomotion activities. To investigate the effect of the ULFMEF-induced optoelectronic stimulation, experimental groups with and without IMER implantation and magnetic field exposure, namely ‘IMER-MF on’, ‘IMER-MF off’, ‘Control-MF on’ and ‘Control-MF off’, were systematically studied and compared in a square activity room (Fig. S24). “MF on” means that there is a magnetic field (MF) covering the IMER, and MF off means the opposite. Furthermore, the multi-angle magnet array was designed for long-distance magnetic energy transfer (Fig. S25). Figure 5k illustrates the typical moving track profile of rats with or without stimulation by the magnetic-field-powered implanted micro-LED integrated IMER device; the optoelectronic stimulation induced by the micro-LED would activate the locomotion behaviors of the rats (Video S3). The experimental results showed the recorded moving tracks (t = 30 minutes) of the rats in the IMER group and control group (without micro-LED integrated IMER implantation) under MF-on and MF-off states (Fig. 5l). When the external rotating magnetic field was applied, the locomotion behaviors of the rats in the IMER group significantly increased due to the optogenetic stimulation, while the rats in the control group exhibited less locomotion. The instantaneous movement velocities within 30 minutes, of the rats in different groups, were analyzed as presented in a heatmap plot and statistically compared (Fig. 5m). The average movement velocity of the IMER group with MF-on was 29.8 mm/s, >3.14-fold higher than the IMER group with MF-off (9.5 mm/s), and >6.47-fold higher than the control groups, respectively. The average locomotion distance and mobility time duration of the rats in different groups were also statistically analyzed (Fig. 5n), and the locomotion distance and mobility time duration increased >6.45-fold and >1.73-fold compared to other control groups. Locomotion behaviors, including traveling distance, mobility time duration and movement velocity, of the rats in the four groups were comprehensively analyzed in a radar plot (Fig. 5o). The results showed that the overall locomotion behaviors of the IMER with MF-on group were significantly enhanced. These results indicated that the rotating magnetic field can effectively power the implanted micro-LED integrated IMER device to generate optogenetic stimulation on motor cortical neurons and activate the rats’ locomotion, demonstrating the potential of ULFMEF to control the behaviors of optogenetic rats through effective wireless powering of implantable devices.

**Figure 6.**
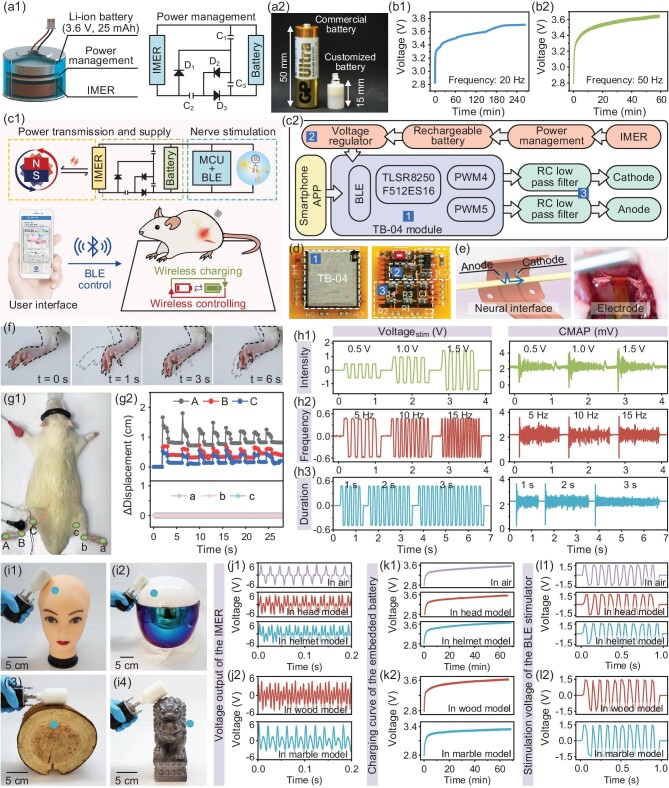
(a1 and a2) Illustration and photograph of the miniature battery-integrated IMER consisting of an IMER, a power management circuit and a rechargeable Li-ion battery, compared with a typical commercial battery. (b1 and b2) Charging curve waveforms of the wireless rechargeable battery at typical rotating frequencies of 20 Hz and 50 Hz. (c1) Illustration of the IMER-based implantable electrical stimulator for modulating neural activity, consisting of the battery-integrated IMER, a micro-control unit, Bluetooth module and flexible nerve-stimulating electrodes. (c2) Detailed block diagram of the wireless bioelectronic stimulator, in which the hardware circuit mainly includes the TB-04 wireless module, power supply module and analog signal output module. (d) Photograph showing the top and bottom layers of the hardware circuit of the implantable IMER electrical stimulator. (e) Illustration and photograph of the flexible nerve-stimulating electrode wrapped on the sciatic nerve for bioelectronic stimulation. (f) Flexion behaviors (t = 1 s, t = 6 s) and extension behaviors (t = 3 s) of the left hind limb in response to ULFMEF-induced electrical stimulation on the rat. (g1 and g2) Movement profiles of the fingertip, elbow and root points at the hind limbs of rats during electrical stimulation. (h1–h3) Output stimulation waveforms with different parameters (intensities, frequencies and durations), and corresponding action potentials of the compound muscle group under different stimulation parameters. (i1–i4) Demonstration of the ULFMEF system powering deeply implanted electronic devices through harder materials, such as silicon rubber, a space suit, wood and marble. (j1 and j2) Typical voltage output waveforms generated by the IMER in different models. (k1 and k2) Wireless charging curve waveforms of the ULFMEF on the implanted battery in different shielding models. (l1 and l2) electrical stimulation outputs of the IMER integrated with the battery and wirelessly controlled by smartphone app in different shielding models.

Implantable electrical stimulators have been shown to be powerful tools for the treatment of many diseases and disorders, such as epilepsy, paralysis and peripheral nerve injuries, through direct electrical stimulation to the target organs [46–48]. Therefore, the ULFMEF methodology was used to wirelessly power an implantable rechargeable battery to provide programmable electrical pulse stimulation on a sciatic nerve *in vivo*. The IMER was integrated with a rechargeable Li-ion battery and a power management circuit (Fig. 6a) to rectify the output alternating current of the IMER into a stable direct current voltage for the Li-ion battery (Fig. 6a1). The miniature battery-integrated IMER was fabricated with a size of Φ 10 × 15 mm^3^ and a weight of only 2.5 g (Fig. 6a2). The type of rechargeable battery is Li-ion 940, with a nominal voltage of 3.6 V and a nominal capacity of 25 mAh. The charging curve waveforms of the wireless rechargeable battery were evaluated, and the Li-ion battery can be charged to ∼3.7 V in 265 minutes (Fig. 6b1) at typical rotating frequencies of 20 Hz, and charged to 3.65 V in 60 minutes at 50 Hz (Fig. 6b2). The voltage points around 3.6 V typically went through the longest duration in the charging cycle. Therefore, the battery was not fully cycled in the experiments, and the charging process was terminated once the voltage reached ∼3.6 V to 3.7 V. When the magnetic field frequency increased, the charging time was shortened at a higher charging rate, due to the increased output current and voltage of IMER with increased magnetic field frequency. An implantable electrical stimulator was further developed (Fig. 6c1) by connecting the battery-integrated IMER with a micro-control unit (MCU), Bluetooth module and flexible nerve stimulating electrodes, which can be wirelessly controlled by smartphone application (app, Fig. S26) for modulating neural activity. When the EMET wirelessly transmitted magnetic energy to the IMER underneath the skin, the induced electricity was charged and stored in the rechargeable battery via the power management circuit. Upon receiving stimulation commands and parameters (including stimulation voltage intensity, frequency and duration) from the smartphone app to the MCU via Bluetooth, the battery-powered MCU generated square-wave voltages for nerve stimulation through a pair of flexible electrodes fixed on the sciatic nerve. The implanted battery can be repeatedly charged wirelessly by the rotating magnetic field. The detailed block diagram of the wireless bioelectronic stimulator is presented in Fig. 6c2. The hardware circuit mainly included a TB-04 wireless module, power supply module and analog signal output module. The TB-04 wireless module enabled wireless data communication with low power consumption and low latency. The power supply module converted the IMER-generated AC signal into a stable DC voltage output, followed by processing by a linear voltage regulator circuit combined with decoupling capacitors to achieve 3.6 V input to 3.3 V output. The voltage can be further adjusted by the pulse-width modulation combined with the resistor-capacitance low-pass filter of the analog signal output module. The top and bottom layers of the hardware circuit of the implantable IMER electrical stimulator are shown in Fig. 6d and Fig. S27. The flexible nerve-stimulating electrodes were surgically implanted and wrapped around the sciatic nerve in the rear leg of the rat (Fig. 6e), forming a stable interface between the nerve and electrodes (anode and cathode, Fig. S28). The implanted electrodes were connected to the power supply part consisting of the battery, IMER and circuit. Notably, this assembly was placed outside the rat's body due to the limited body size of the rat in this proof-of-concept experiment. After being triggered by the app command, the device output electricity to the sciatic nerve via the implanted electrodes, resulting in the rhythmical movement of the hind limb. Electrical stimulation of the sciatic nerve fibers may induce neural action potentials that trigger the crossing-bridge cycle and cause contractions and relaxations in gastrocnemius muscle, which is the basis of many rehabilitation and neuromodulation methods. The IMER can generate programmed stimulation voltages (∼1.5 V and ∼10 Hz) on the sciatic nerve. When the electrical stimulation was applied, the muscles of the hind limbs contracted in response to the stimulation. Obvious flexion behavior (t = 1 s) of the left hind limb can be observed. When the stimulation voltage was applied again, another cycle of extension (t = 3 s) to flexion (t = 6 s) behavior of the hind limb can be consistently observed upon the electrical stimulation of the sciatic nerve (Fig. 6f, Fig. S29 and Video S2). The results showed that the hind limbs of the rats can readily and repeatedly respond to ULFMEF-induced electrical stimulation even after multiple cycles of tests. The movement profiles of the fingertip, elbow and root (indicated as point A, B, C in Fig. 6g1) on the hind limbs of rats during the electrical stimulation were tracked and analyzed. The left limb, implanted with an electrode, exhibited rhythmic flexion and extension in response to electrical stimulation, where the maximum displacement of the fingertip was ∼1 cm, while the right limb without electrode implantation showed no obvious movement (Fig. 6g2). Meanwhile, the action potentials of the compound muscle group under different stimulation parameters (intensities, frequencies and durations, Fig. S30) were recorded (Fig. 6h). The fluctuation of muscle group action potentials can be clearly observed upon electrical stimulation of the sciatic nerve. When the intensities of stimulation voltages increased from 0.5 V to 1.5 V (Fig. 6h1), the peak-peak action potentials of the compound muscle group increased from 3.55 mV to 5.5 mV, resulting in stronger beats of the rear leg. The rear legs synchronously swung with a stimulation frequency ranging from 5 Hz to 15 Hz (Fig. 6h2), and the induced action potentials of the muscle group were also observed to continuously fluctuate throughout electrical stimulation periods when the stimulation duration was increased from 1 s to 3 s (Fig. 6h3). The above experiments demonstrated that the ULFMEF can wirelessly power an IMER coupled to an integrated battery and circuit to provide programmable electrical stimulation on nerves *in vivo*, suggesting the versatility of ULFMEF for complex biomedical applications. While using the ULFMEF technique, backscatter communication is difficult to achieve, which restricts the perception and feedback of neuroelectric stimulation.

The above experiments illustrated the ability of the ULFMEF system to power implantable electronic devices through soft biological tissues. Furthermore, it was demonstrated that the wireless energy transfer of ULFMEF can even effectively penetrate harder materials, such as silicon rubber, a space suit, wood or marble to power electronic devices at deeper depths (∼3 cm to 10 cm) (Fig. 6i). Specifically, the IMER was implanted in a rubber model of a head, at a depth of ∼3 cm (Fig. 6i1), then with the head wearing a spacesuit helmet (thickness ∼1.5 cm, Fig. 6i2). The IMER was also implanted in wood at a depth of ∼5 cm (Fig. 6i3), and in a marble model at a depth of 10 cm (Fig. 6i4), respectively. The voltage output performance, battery charging capability and programmed stimulation voltage outputs of the IMER embedded in different materials were tested, while the electrical output in air was employed for comparison. The typical voltage output waveforms generated by the IMER in different models are shown in Fig. 6j. At 50 Hz ULFMEF operation, the IMER can generate voltage in the range of 4.5–5.6 V for the spacesuit, wood and marble models. This closely approximated the electrical output in air, indicating that the magnetic field can penetrate the mentioned solid materials to generate considerable electrical output almost without loss. The wireless charging ability of the ULFMEF on the implanted battery also showed the increased voltage of the battery within one hour during the charging process (Fig. 6k). After 70 minutes, the voltage of the Li-ion battery can be charged to ∼3.3 V to 3.6 V, whereupon the charging curves of different shielding models were similar to that in air, indicating that the shielding material did not prevent the rotating magnetic field from powering the IMER to charge the battery. Finally, the electrical stimulation output (Fig. 6l) of the IMER integrated with a battery and wireless control by smartphone app were also evaluated in the above different shielding models. The IMER-integrated stimulator embedded in the above models can generate corresponding stimulation voltages (amplitude of 1.5 V and frequency of 10 Hz) according to the commands of the app, which indicated that the above shielding materials did not obstruct wireless power and information transmission between the implanted stimulator and the external smartphone device. The above results demonstrated that the ULFMEF possessed excellent wireless energy transfer that penetrated thick and tough solid materials. This would make it possible to wirelessly power implantable devices for humans wearing special suits, such as protective suits or spacesuits in aerospace or military scenarios; these types of scenarios were challenging for conventional wireless powering technologies based on near-infrared light, ultrasound or electromagnetic waves.

## CONCLUSIONS

In this study, we proposed an ultra-low-frequency magnetic energy transfer system with considerable electrical output performance, low energy attenuation, long-distance energy transmission, limited heat generation and good biosafety. This system consists of a portable magnetic energy transmitter and a miniature IMER with a small size of Φ 8 × 4 mm^3^. The ULFMEF can output considerable electricity with a maximum output power of ∼9.35 mW, a peak voltage of 3 V and a peak current of 15 mA under a rotating magnetic field with a low frequency of 20 Hz and transmission distance of 5 cm. Under a 20-Hz rotating magnetic field, the maximum energy transmission distance of this system can reach ∼35 cm, and the electrical output of the IMER exhibited a nearly linear attenuation (38% power attenuation from 5 cm to 10 cm). The IMER can maintain operation for 30 minutes with a temperature rise of <0.5°C, exhibiting low heat generation and limited potential risk of thermal damage to the tissue. Moreover, this study experimentally demonstrated that the IMER created limited cytotoxicity of cells and visceral tissues. Based on the IMER, a battery-free optoelectronic stimulator and a smartphone-based nerve bioelectronic stimulator were developed. The optoelectronic stimulators implanted in the heads of optogenetic rats can manipulate their behaviors in a low magnetic field frequency. The nerve bioelectronic stimulator implanted in the rear leg of a rat can wirelessly stimulate the sciatic nerve. Therefore, because of its unique advantages, the ultra-low frequency magnetic energy transmission system has promising applications in wireless energy transmission for medical implants, such as cardiac pacemakers, spinal cord stimulators and deep-brain stimulators. We designed a bedplate for the wireless charging of medical implants, which not only eliminated the potential risk inherent in battery replacement surgeries but also provided great freedom for patients (Fig. S31).

## ETHICAL STATEMENTS

Ethical permission was granted by the School of Medicine, Zhejiang University. Informed consent was obtained from all donors.

## Supplementary Material

nwae062_Supplemental_Files

## References

[1] Choi YS, Jeong H, Yin RT et al. A transient, closed-loop network of wireless, body-integrated devices for autonomous electrotherapy. Science 2022; 376: 1006–12. 10.1126/science.abm1703 35617386 PMC9282941

[2] Yin RT, Chen SW, Lee KB et al. Open thoracic surgical implantation of cardiac pacemakers in rats. Nat Protoc 2023; 18: 374–95. 10.1038/s41596-022-00770-y 36411351 PMC11939016

[3] Meng K, Xiao X, Liu Z et al. Kirigami-inspired pressure sensors for wearable dynamic cardiovascular monitoring. Adv Mater 2022; 34: 2202478. 10.1002/adma.202202478 35767870

[4] Hoare D, Tsiamis A, Marland JRK et al. Predicting cardiovascular stent complications using self-reporting biosensors for noninvasive detection of disease. Adv Sci 2022; 9: 2105285. 10.1002/advs.202105285 PMC9130883 35322587

[5] Zaeimbashi M, Nasrollahpour M, Khalifa A et al. Ultra-compact dual-band smart NEMS magnetoelectric antennas for simultaneous wireless energy harvesting and magnetic field sensing. Nat Commun 2021; 12: 3141. 10.1038/s41467-021-23256-z 34035237 PMC8149822

[6] Liang L, Liu C, Cai P et al. Highly specific differentiation of MSCs into neurons directed by local electrical stimuli triggered wirelessly by electromagnetic induction nanogenerator. Nano Energy 2022; 100: 107483. 10.1016/j.nanoen.2022.107483

[7] Ryu H, Park H-M, Kim M-K et al. Self-rechargeable cardiac pacemaker system with triboelectric nanogenerators. Nat Commun 2021; 12: 4374. 10.1038/s41467-021-24417-w 34272375 PMC8285394

[8] Choi YS, Yin RT, Pfenniger A et al. Fully implantable and bioresorbable cardiac pacemakers without leads or batteries. Nat Biotechnol 2021; 39: 1228–38. 10.1038/s41587-021-00948-x 34183859 PMC9270064

[9] Yang YX, Qiao SY, Sani OG et al. Modelling and prediction of the dynamic responses of large-scale brain networks during direct electrical stimulation. Nat Biomed Eng 2021; 5: 324–45. 10.1038/s41551-020-00666-w 33526909

[10] Hescham S-A, Chiang P-H, Gregurec D et al. Magnetothermal nanoparticle technology alleviates parkinsonian-like symptoms in mice. Nat Commun 2021; 12: 5569. 10.1038/s41467-021-25837-4 34552093 PMC8458499

[11] Kim CY, Ku MJ, Qazi R et al. Soft subdermal implant capable of wireless battery charging and programmable controls for applications in optogenetics. Nat Commun 2021; 12: 535. 10.1038/s41467-020-20803-y 33483493 PMC7822865

[12] Rajalingham R, Sorenson M, Azadi R et al. Chronically implantable LED arrays for behavioral optogenetics in primates. Nat Methods 2021; 18: 1112–6. 10.1038/s41592-021-01238-9 34462591

[13] Ma C, Ni XY, Zhang YQ et al. Implanting an ion-selective “skin” in electrolyte towards high-energy and safe lithium-sulfur battery. Matter 2022; 5: 2225–37. 10.1016/j.matt.2022.04.017

[14] Simons P, Schenk SA, Gysel MA et al. A ceramic-electrolyte glucose fuel cell for implantable electronics. Adv Mater 2022; 34: 2109075. 10.1002/adma.202109075 35384081

[15] Han S, Sung W, Kim TY et al. Upconversion nanoparticles coated organic photovoltaics for near infrared light controlled drug delivery systems. Nano Energy 2021; 81: 105650. 10.1016/j.nanoen.2020.105650

[16] Wu WX, Guo NY, Li W et al. The vitro/vivo anti-corrosion effect of antibacterial irTENG on implantable magnesium alloys. Nano Energy 2022; 99: 107397. 10.1016/j.nanoen.2022.107397

[17] Iacovacci V, Tamadon I, Kauffmann EF et al. A fully implantable device for intraperitoneal drug delivery refilled by ingestible capsules. Sci Robot 2021; 6: 3328. 10.1126/scirobotics.abh3328 34408097

[18] Wu QN, Yang C, Chen W et al. Wireless-powered electrical bandage contact lens for facilitating corneal wound healing. Adv Sci 2022; 9: 2202506. 10.1002/advs.202202506 PMC9631068 36073832

[19] Mickle AD, Won SM, Noh KN et al. A wireless closed-loop system for optogenetic peripheral neuromodulation. Nature 2019; 565: 361–5. 10.1038/s41586-018-0823-6 30602791 PMC6336505

[20] Jiang DJ, Shi BJ, Ouyang H et al. Emerging implantable energy harvesters and self-powered implantable medical electronics. ACS Nano 2020; 14: 6436–48. 10.1021/acsnano.9b08268 32459086

[21] Xue HY, Jiang LM, Lu GX et al. Multilevel structure engineered lead-free piezoceramics enabling breakthrough in energy harvesting performance for bioelectronics. Adv Funct Mater 2023; 33: 2212110. 10.1002/adfm.202212110

[22] Liu XZ, Wang YQ, Wang GY et al. An ultrasound-driven implantable wireless energy harvesting system using a triboelectric transducer. Matter 2022; 5: 4315–31. 10.1016/j.matt.2022.08.016

[23] Hu F, Li WF, Zou MY et al. Subcutaneous energy/signal transmission based on silk fibroin up-conversion photonic amplification. ACS Nano 2021; 15: 9559–67. 10.1021/acsnano.0c09575 33382583

[24] Song K, Han JH, Lim T et al. Subdermal flexible solar cell arrays for powering medical electronic implants. Adv Healthcare Mater 2016; 5: 1572–80. 10.1002/adhm.201600222 27139339

[25] Liu HZ, Zhao TT, Jiang WT et al. Flexible battery-less bioelectronic implants: wireless powering and manipulation by near-infrared light. Adv Funct Mater 2015; 25: 7071–9. 10.1002/adfm.201502752

[26] Yamagishi K, Zhou WS, Ching T et al. Ultra-deformable and tissue-adhesive liquid metal antennas with high wireless powering efficiency. Adv Mater 2021; 33: 2008062. 10.1002/adma.202008062 34031936

[27] Saito M, Kanai E, Fujita H et al. Flexible induction heater based on the polymeric thin film for local thermotherapy. Adv Funct Mater 2021; 31: 2102444. 10.1002/adfm.202102444

[28] Kwon K, Kim JU, Won SM et al. A battery-less wireless implant for the continuous monitoring of vascular pressure, flow rate and temperature. Nat Biomed Eng 2023; 7: 1215–28. 10.1038/s41551-023-01022-4 37037964

[29] Yang S-Y, Sencadas V, You SS et al. Powering implantable and ingestible electronics. Adv Funct Mater 2021; 31: 2009289. 10.1002/adfm.202009289 34720792 PMC8553224

[30] Yuan ZH, Jin X, Li RN et al. Hybrid triboelectric-electromagnetic magnetic energy harvester-based sensing for wireless monitoring of transmission lines. Small 2022; 18: 2107221. 10.1002/smll.202107221 35678105

[31] Zheng JY, Cao Z, Han C et al. A hybrid triboelectric-electromagnetic nanogenerator based on arm swing energy harvesting. Nanoenergy Adv 2023; 3: 126–37. 10.3390/nanoenergyadv3020007

[32] Wu ZY . Triboelectric nanogenerator for human-machine interfacing. In: Wang ZL, Yang Y, Zhai JY, Wang J (eds). Handbook of Triboelectric Nanogenerators. Cham: Springer, 2023, 1591–619.

[33] Lyu SZ, He YL, Tao XL et al. Subcutaneous power supply by NIR-II light. Nat Commun 2022; 13: 6596. 10.1038/s41467-022-34047-5 36329024 PMC9633840

[34] Jeong J, Jung J, Jung D et al. An implantable optogenetic stimulator wirelessly powered by flexible photovoltaics with near-infrared (NIR) light. Biosens Bioelectron 2021; 180: 113139. 10.1016/j.bios.2021.113139 33714161

[35] Wan X, Chen P, Xu ZS et al. Hybrid-piezoelectret based highly efficient ultrasonic energy harvester for implantable electronics. Adv Funct Mater 2022; 32: 2200589. 10.1002/adfm.202200589

[36] Piech DK, Johnson BC, Shen K et al. A wireless millimetre-scale implantable neural stimulator with ultrasonically powered bidirectional communication. Nat Biomed Eng 2020; 4: 207–22. 10.1038/s41551-020-0518-9 32076132

[37] Hinchet R, Yoon H-J, Ryu H et al. Transcutaneous ultrasound energy harvesting using capacitive triboelectric technology. Science 2019; 365: 491–4. 10.1126/science.aan3997 31371614

[38] Bansal A, Yang FY, Xi T et al. *In vivo* wireless photonic photodynamic therapy. Proc Natl Acad Sci USA 2018; 115: 1469–74. 10.1073/pnas.1717552115 29378941 PMC5816188

[39] Sun B, Bte Rahmat JN, Kim HJ et al. Wirelessly activated nanotherapeutics for *in vivo* programmable photodynamic-chemotherapy of orthotopic bladder cancer. Adv Sci 2022; 9: 2200731. 10.1002/advs.202200731 PMC9165499 35393785

[40] Chen JC, Kan P, Yu ZH et al. A wireless millimetric magnetoelectric implant for the endovascular stimulation of peripheral nerves. Nat Biomed Eng 2022; 6: 706–16. 10.1038/s41551-022-00873-7 35361934 PMC9213237

[41] Lee HE, Park JH, Jang D et al. Optogenetic brain neuromodulation by stray magnetic field via flash-enhanced magneto-mechano-triboelectric nanogenerator. Nano Energy 2020; 75: 104951. 10.1016/j.nanoen.2020.104951

[42] Guo QL, Koo J, Xie ZQ et al. A bioresorbable magnetically coupled system for low-frequency wireless power transfer. Adv Funct Mater 2019; 29: 1905451. 10.1002/adfm.201905451

[43] Yang YY, Wu MZ, Vazquez-Guardado A et al. Wireless multilateral devices for optogenetic studies of individual and social behaviors. Nat Neurosci 2021; 24: 1035–45. 10.1038/s41593-021-00849-x 33972800 PMC8694284

[44] Zhou LP, Zhang YZ, Cao G et al. Wireless self-powered optogenetic system for long-term cardiac neuromodulation to improve post-mi cardiac remodeling and malignant arrhythmia. Adv Sci 2023; 10: 2205551. 10.1002/advs.202205551 PMC10037959 36698262

[45] Chen P, Wu P, Wan X et al. Ultrasound-driven electrical stimulation of peripheral nerves based on implantable piezoelectric thin film nanogenerators. Nano Energy 2021; 86: 106123. 10.1016/j.nanoen.2021.106123

[46] Yang HR, Su Y, Sun ZY et al. Gold nanostrip array-mediated wireless electrical stimulation for accelerating functional neuronal differentiation. Adv Sci 2022; 9: 2202376. 10.1002/advs.202202376 PMC9353484 35618610

[47] Kaliannagounder VK, Raj NPMJ, Unnithan AR et al. Remotely controlled self-powering electrical stimulators for osteogenic differentiation using bone inspired bioactive piezoelectric whitlockite nanoparticles. Nano Energy 2021; 85: 105901. 10.1016/j.nanoen.2021.105901

[48] Cai L, Burton A, Gonzales DA et al. Osseosurface electronics-thin, wireless, battery-free and multimodal musculoskeletal biointerfaces. Nat Commun 2021; 12: 6707. 10.1038/s41467-021-27003-2 34795247 PMC8602388

