# Supplementary material for: Ultra-low frequency magnetic energy focusing for highly effective wireless powering of deep-tissue implantable electronic devices
Source: Natl Sci Rev. 2024 Feb 28;11(5):nwae062. doi: 10.1093/nsr/nwae062 (PMC11020258; doi:10.1093/nsr/nwae062)
Supplement: nwae062_Supplemental_Files [file nwae062_supplemental_files.zip › Supplementary data.pdf]

Supplementary materials for

Ultra-low frequency magnetic energy focusing for highly effective wireless-powering of deep-tissue implantable electronic devices

Yuanyuan Li[a,#], Zhipeng Chen[a,b,#], Yuxin Liu[a], Zijian Liu[a], Tong Wu[a], Yuanxi Zhang[a], Lelun Peng[a], Xinshuo Huang[c], Shuang Huang[c], Xudong Lin[a], Xi Xie[a,c,*] and Lelun Jiang[a,*]

[a]Guangdong Provincial Key Laboratory of Sensor Technology and Biomedical Instrument; School of Biomedical Engineering, Shenzhen Campus of Sun Yat-Sen University, Shenzhen 518107, China;

[b]School of Mechanical and Electrical Engineering, Guangzhou University, Guangzhou 510006, China;

[c]State Key Laboratory of Optoelectronic Materials and Technologies; Guangdong Province Key Laboratory of Display Material and Technology; School of Electronics and Information Technology, Sun Yat-Sen University, Guangzhou 510006, China

[*]**Corresponding authors**. E-mails: jianglel@mail.sysu.edu.cn; xiexi27@mail.sysu.edu.cn

[#]Equally contributed to this work.

1. Design and fabrication of the implantable magnetic energy receiver (IMER)

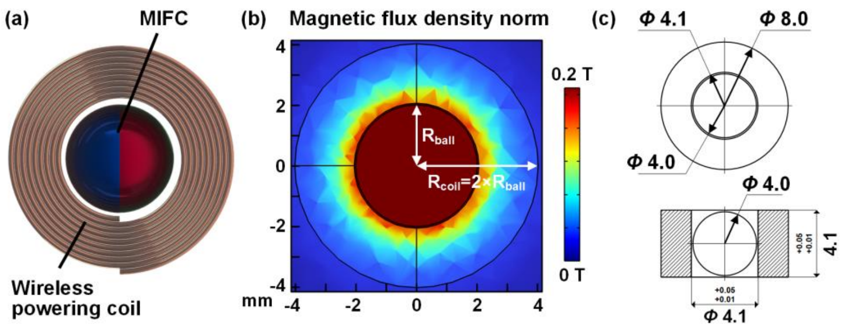

Figure S1. (a) Schematic diagram of the IMER. **(b)** Magnetic field distribution of the IMER. **(c)** Engineering schematics of the IMER.

The implantable IMER was designed as shown in Figure S1. The IMER was mainly composed of the wireless powering coil and the magnetism internally focusing core (MIFC) (Figure S1a). The magnetic field distribution of IMER generated by the MIFC was analyzed using COMSOL Multiphysics 6.0 (COMSOL Inc., Sweden) (Figure S1b). The magnetic field distribution of the magnetic core gradually attenuated along the radial direction. The magnetic field intensity was very low where the radial distance was beyond twice the ball radius from its center. Therefore, the outer radius of the copper coil was designed to be twice of ball radius to minimize the IMER size and make the most of this magnetic field. The detailed design of IMER with the size of $\Phi\ 8\times4\ mm^3$ was shown in Figure S1c. The diameter of the MIFC was 4 mm. The outer diameter of the coil was 8 mm, and the height of the coil was 4.1 mm. The spherical surface of the MIFC was in clearance fit with the inner surface of the cylinder coil to minimize the friction between the MIFC and coil during the rotation driven by the magnetic field. The wire diameter of the copper coil was designed as 0.06 mm, which could be adjusted according to the actual demand.

2. Magnetic flux density of the magnetic core

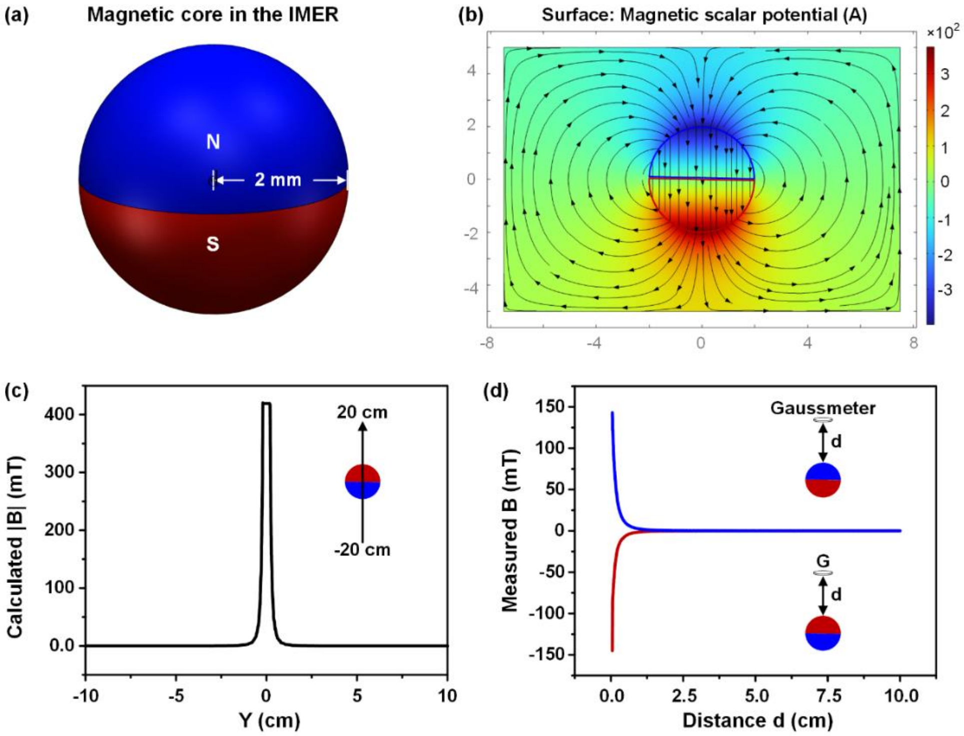

Figure S2. (a) Geometry of the magnetic core. **(b)** Calculated flux density distribution of the magnetic core. **(c)** Calculated flux density mode of the magnetic core under different distances. **(d)** Experimentally measured flux density of the magnetic core under different distances.

The magnetic flux density of the magnetic core in the IMER was calculated by FEA and experimental measured, as shown in Figure S2. In the simulation, the surface magnetic flux density of the magnetic core was 419 mT, and when the distance increased to 5 cm, the magnetic flux density modes were about 0.2 mT. In the experiment, the surface magnetic flux density of the magnetic core was 144 mT (), and when the distance increased to 5 cm, the magnetic flux density modes were about 0.1 mT. The surface magnetic flux densities of the magnets were measured using a digital Gauss meter (CH 1500, CH-Magnetoelectricity Technology, China).

3. Design of the external magnetic energy transmitter

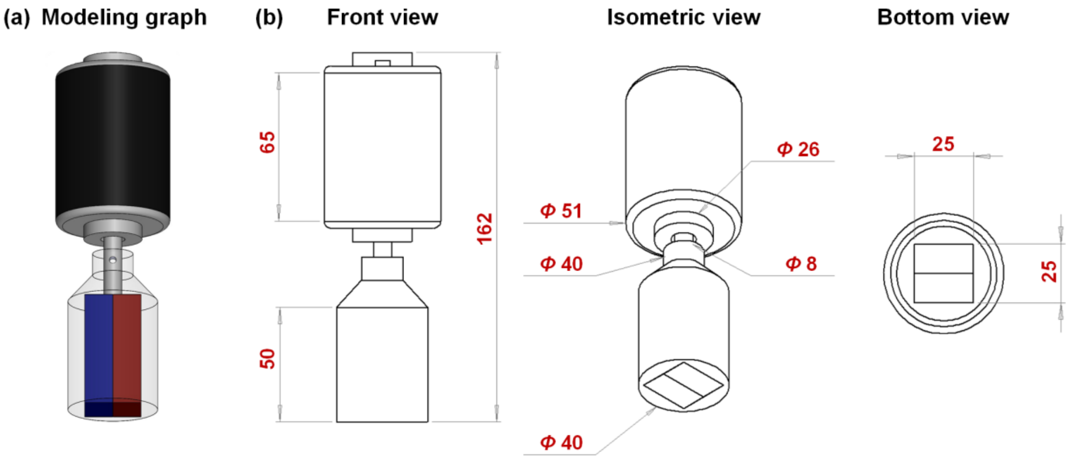

Figure S3. (a) Design illustration of the external magnetic energy transmitter. **(b)** The engineering schematics (front view, isometric view and bottom view) showing the actual dimensions of the EMET. The unit: mm.

The external magnetic energy transmitter (EMET) for wireless powering was designed as shown in Figure S3. The transmitter simply consisted of a NdFeB magnet (N42, 50×25×25 mm3) and a 24V-DC motor (XD3420, XIN DA MOTOR CO., LTD, China). The portable transmitter could generate a rotating magnetic field at ultra-low frequency of 5-100 Hz tuned by a speed controller. The engineering schematics, including front view, isometric view and bottom view, showed the actual dimensions of the EMET. The total length of the EMET was 162 mm, which was portable and convenient for self-operation by patient in daily life.

4. Magnetic flux density of the driving magnet

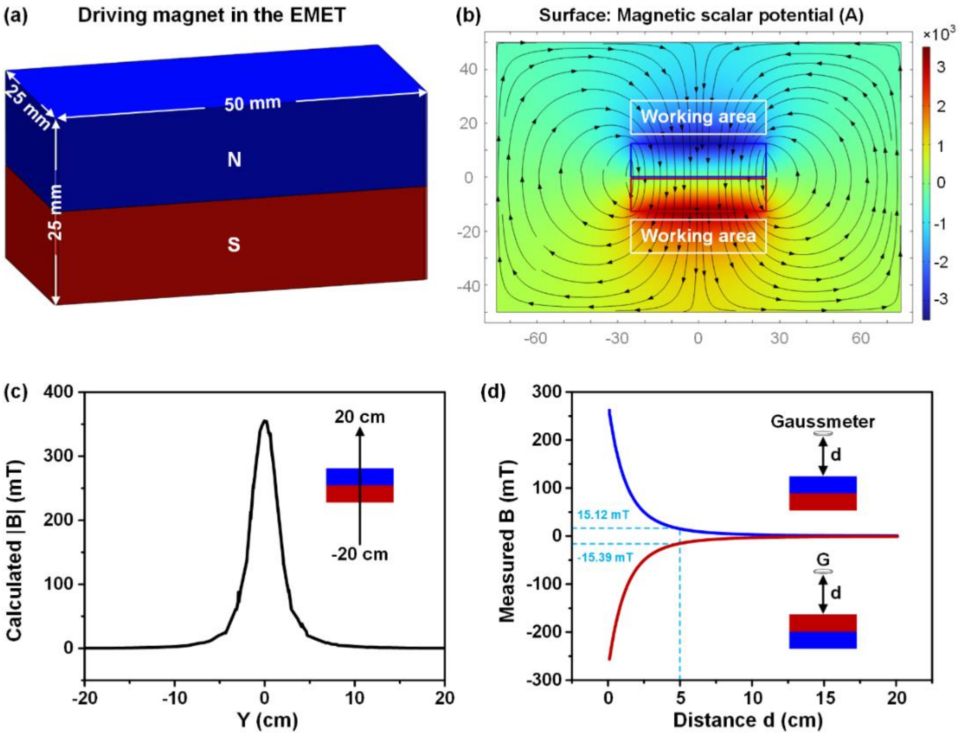

Figure S4. (a) Geometry of the driving magnet. **(b)** Calculated flux density distribution of the driving magnet. **(c)** Calculated flux density mode of the driving magnet under different distances. **(d)** Experimentally measured flux densities of the driving magnet with distance.

The magnetic flux density of the driving magnet in the EMET was investigated by simulation calculations and experimental measurements, as shown in Figure S4. In the simulation, the surface magnetic flux density of the driving magnet is 355 mT, and when the distance increased to 5 cm and 10 cm, the magnetic flux density modes were about 18 mT and 3.2 mT, respectively. In the experiment, the surface magnetic flux density of the driving magnet is 260 mT, and when the distance increased to 5 cm and 10 cm, the magnetic flux density modes were about 15 mT and 3 mT, respectively.

5. Fabrication of the IMER

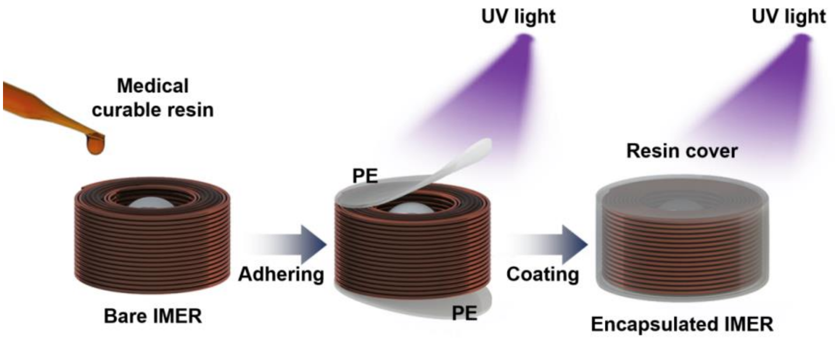

Figure S5. The fabrication process of the IMER.

The fabrication process of the IMER was shown in Figure S5. Firstly, the MIFC was assembled in the cylinder coil. Then two sides of the cylinder coil were sealed with two transparent polyethylene sheets (PE sheets, diameter: 8 mm and thickness: 160 μm, Runwen Company) and glued with the medical photopolymer (Flashforge, FHD 1500) under UV radiation. Finally, the surface of IMER was uniformly coated with the medical photopolymer film to enhance hermeticity and biocompatibility. A series of IMERs with the size of Φ 6×3 mm^3, Φ 8×4 mm^3, and Φ 10×5 mm^3 with the MIFC diameter of 3 mm, 4 mm, and 5 mm were fabricated, respectively.

6. SEM image of the IMER surface

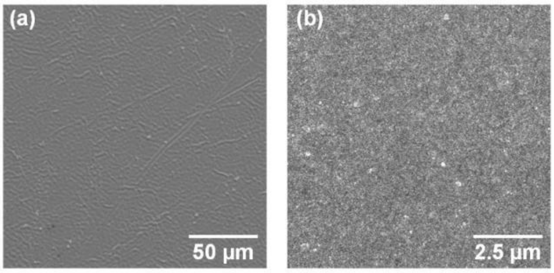

Figure S6. SEM images of IMER surface coated with medical photopolymer.

The IMER surface coated with medical photopolymer was observed using the scanning electron microscope (SEM, JSM-6380LA, JEOL, Japan). The IMER was uniformly sealed with medical photosensitive resin to effectively avoid infiltration and corrosion by biological tissue fluid during implantation, thereby improving its biocompatibility. The surface of IMER was extremely smooth without any pores, demonstrating good anti-permeability performance, which was necessary for implantable electronic devices.

7. Magnetic force strength between the EMET and the IMER

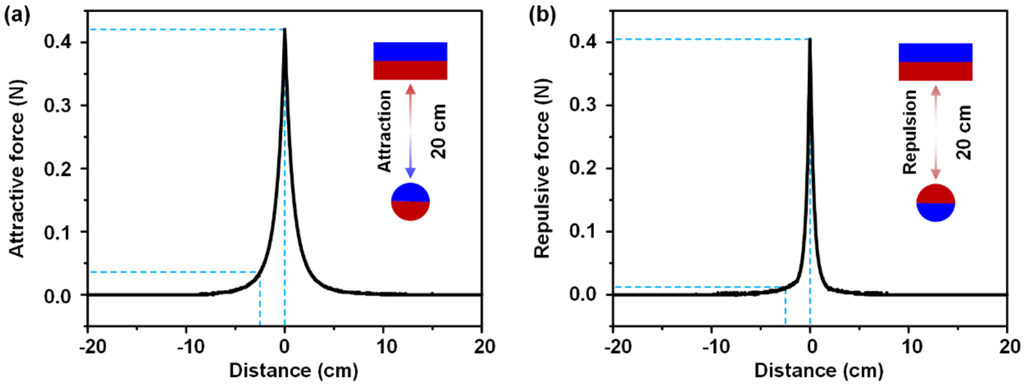

Figure S7. (a) Magnetic attraction force and **(b)** magnetic repulsion force between driving magnet and magnetic core under different distances.

The magnetic force strength between the driving magnet in the EMET and the magnetic core in the IMER was investigated by experimental measurements. When the distance between the driving magnet and the magnetic core was 0.5 cm, the magnetic attraction force between them was ~421 mN and the magnetic repulsion force was ~398 mN. While when the distance increased to 5 cm, the magnetic attraction force was about 7.5 mN and the magnetic repulsion force was about 2 mN, and when the distance was raised to 10 cm, the magnetic attraction force decreased to 0.5 mN.

8. Analysis of ultra-low frequency magnetic energy focusing (ULFMEF)

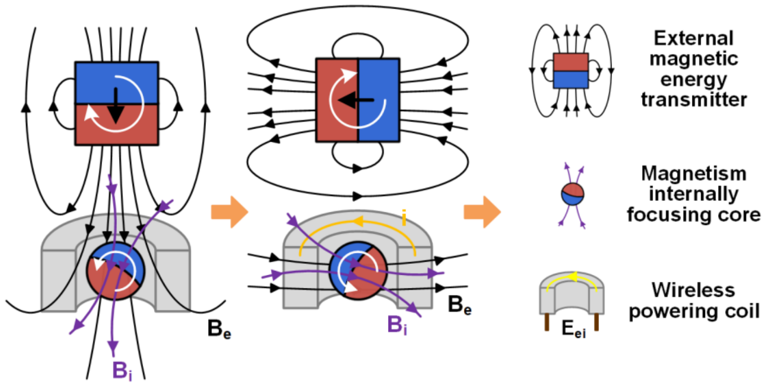

Figure S8. Schematic diagram of magnetic induction mechanism of the ULFMEF system.

The wireless magnetic energy focusing mechanism of this system was analyzed in Figure 1. Based on Faraday's law of electromagnetic induction, the rotating magnetic field generates the changing magnetic flux and induces the electric potential in the coil. In particular, the induced electromotive force generated in the IMER consists of two components: Component 1 attributing to the rotation of the external magnetic energy transmitter, and Component 2 owing to the rotation of the MIFC. A continuous and stable electric output can be produced in the coil, and the induced current I can be calculated according to Faraday's law of electromagnetic induction:

$$I = \frac{E}{R} = -NS\frac{d\Phi}{dt}\frac{1}{R} = -\frac{NS}{R}\frac{d(B_i\cos\theta + \overline{B_e}\cos\varphi)}{dt} \quad \text{(S1)}$$

When the magnetic energy transmission process is in a dynamic equilibrium state, it is considered that the MIFC and the driving magnet rotated at the same frequency with a fixed average phase difference δ. Therefore, the rotation angle of the driving magnet is $\varphi = \omega t + \delta$, and the rotation angle of the MIFC is $\theta = \omega t$, where the ω is the rotating speed of the magnetic field, and the phase difference δ is determined by the sum function of the magnetic force, coil resistance, and friction force. Equation (S1) can be approximated as:

$$I(t) = -\frac{NS}{R}\frac{d}{dt}[B_{\mathrm{i}}\cos(\omega t) + \overline{B_{\mathrm{e}}}\cos(\omega t + \delta)] \quad \text{(S2)}$$

Based on the trigonometric identity, Equation (S2) can be transformed as:

$$I(t) = -\frac{NS}{R}\sqrt{B_{\mathrm{i}}^2 + \overline{B_{\mathrm{e}}}^2 + 2B_{\mathrm{i}}\overline{B_{\mathrm{e}}}cos\delta} \cdot \frac{d}{dt}\sin[\omega t + \arctan\left(-\frac{B_{\mathrm{i}} + \overline{B_{\mathrm{e}}}\cos\delta}{\overline{B_{\mathrm{e}}}\sin\delta}\right)] \quad \text{(S3)}$$

As the transmission in a dynamic equilibrium state, Equation (S3) can be evolved as:

$$I(t) = -\frac{NS}{R}\sqrt{B_{\mathrm{i}}^2 + \overline{B_{\mathrm{e}}}^2 + 2B_{\mathrm{i}}\overline{B_{\mathrm{e}}}cos\delta} \cdot \omega \cdot \cos[\omega t + \arctan\left(-\frac{B_{\mathrm{i}} + \overline{B_{\mathrm{e}}}\cos\delta}{\overline{B_{\mathrm{e}}}\sin\delta}\right)] \quad \text{(S4)}$$

It can be inferred that the induced current I(t) of the IMER is the cosine function. It is also proportional to coil turns N, the cross-sectional area of the coil S, and the rotating speed of the magnetic field ω while inversely proportional to the coil resistance R. I(t) is also positively related to the magnetic field intensity of the MIFC B_{i} and the average magnetic field intensity of the driving magnet on the IMER $\overline{B_{\mathrm{e}}}$. When the IMER is close to the driving magnet, $\overline{B_{\mathrm{e}}}$ is much larger than B_{i}, and I(t) is mainly determined by $\overline{B_{\mathrm{e}}}$, which can be tuned by the field intensity of the driving magnet. Nevertheless, if the IMER is far away from the driving magnet, B_{i} is much larger than $\overline{B_{\mathrm{e}}}$, and I(t) is mainly determined by B_{i}, which is magnetic field intensity of the MIFC. Therefore, it can be seen from the theoretical derivation that the electric output of the IMER driven by the ultra-low frequency rotating magnetic field can be tuned by above parameters.

9. Finite element analysis (FEA) of magnetic energy transmission

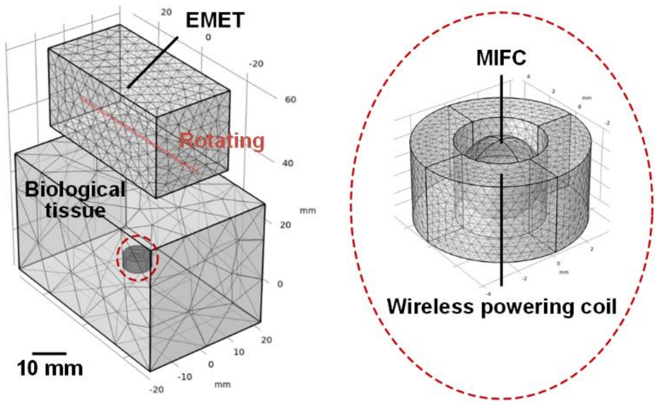

Figure S9. FEA of the wireless magnetic energy transmission system.

Finite element analysis (FEA) was employed to analyze the external magnetic energy transmission of the system using COMSOL Multiphysics 6.0 (COMSOL Inc., Sweden). The multi-physics model of

FEA was built in a spheriform space (diameter: 100 cm), which mainly consisted of the air, driving magnet, MIFC, and wireless powering coil. The geometric models of the parts were built based on their actual sizes. The geometric size of the driving magnet was set as $50\times25\times25$ mm^3. The diameter of the spherical MIFC was set as 4 mm. The external diameter, internal diameter, and height of the coil were set as 8 mm, 4.1 mm, and 4.1 mm, respectively. In azimuth, the distance between the IMER and the driving magnet along the Z-axis was 5 cm. The models meshed with tetrahedral elements in extremely fine. The magnetic fields of EMET and the MIFC in IMER, B_e and B_i, were assumed with the strength of ~700-1100 kA/m, which rotate synchronously at a uniform frequency (frq) in the range of 10-50 Hz. The material of the coil was set as copper, and its relative permeability and permittivity were set as 1.0 and 1.0, respectively.

The magnetic energy transmission process was analyzed using the “Magnetic Field” module, where the magnetic field distribution and electrical outputs were calculated, respectively. Firstly, the time-varying magnetic field distribution was calculated using “Ampere's law” in time-dependent. The rotation axis of both the driving magnet and the MIFC were parallel to the X-axis with a rotation speed of 32 r/s, and the rotation direction of the MIFC is opposite to that of the driving magnet. Secondly, the voltage and current intensity generated by the IMER were calculated using “Coil”. The conductor model was set as “Homogenized multiturn”. The coil type was set as “Circular”. The coil excitation was set as “Voltage” or “Current”. About the homogenized multiturn conductor, the number of turns was set as 3800. The coil wire conductivity was set as 6×10^7 S/m. The wire diameter of the coil was set as 0.06 mm. Then the above calculations were performed. The 3D plot group was used to depict the magnetic field intensity distribution of the system, and the 1D plot group was used to show the induced voltage or induced current.

10. Magnetic field distribution of the driving magnet

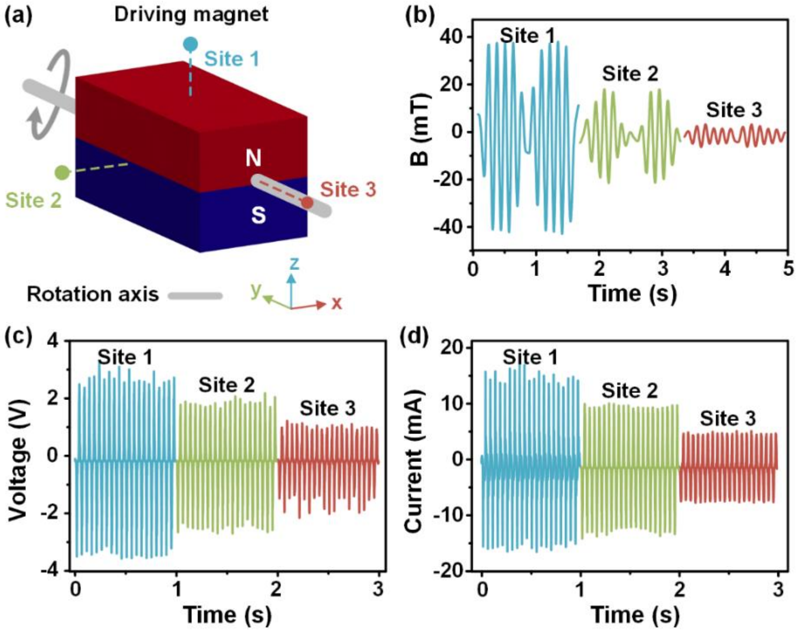

Figure S10. **(a)** Schematic diagram of the driving magnet. **(b)** Magnetic field intensity of the driving magnet at different sites. **(c)** The output voltage and (d) current of IMER at site 1, site 2, and site 3.

The magnetic field distribution of the driving magnet had a significant effect on the electric output

performance of the IMER. Therefore, the magnetic field intensity and the electric output performance of the IMER at three different axes (site 1 on X-Y plane, site 2 on Y-Z plane, and site 3 on X-Z plane with a distance of 5 cm from the magnet surface) (Figure S10a) were measured. The magnetic field intensity generated by the driving magnet at site 1, site 2, and site 3 were 40 mT, 20 mT, and 5 mT, respectively (Figure S10b). The output electricity of IMER was tested using an electrometer (6514, Keithley, USA). The output open-circuit voltage (V_{oc}) of IMER working at site 1, site 2, and site 3 were 3 V, 2 V, and 1.2 V, respectively (Figure S10c). The output short circuit currents of IMER working at site 1, site 2, and site 3 were 16 mA, 11.5 mA, and 6 mA, respectively (Figure S10d). Therefore, the IMER output the maximum electricity along the z-axis magnetic direction on the X-Y plane. Therefore, the external/internal magnetism synergistically-generated electricity was measured along the z-axis direction of the driving magnet.

11. LEDs lightened by the ULFMEF system

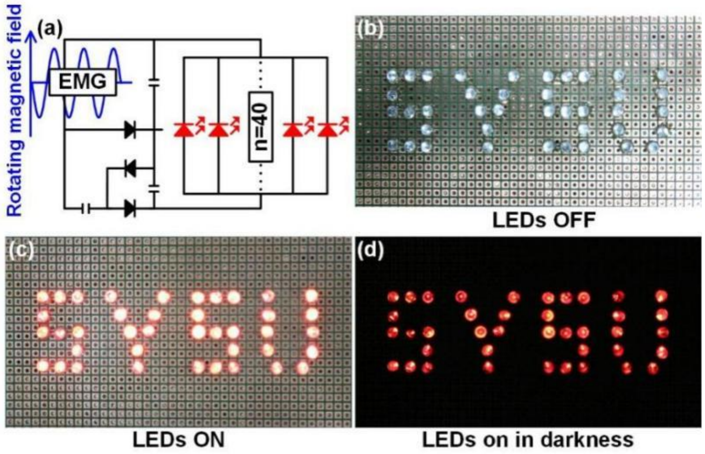

Figure S11. The LEDs in parallel were lightened by the ULFMEF system.

The IMER, power management circuit, and LEDs were connected as Figure S11a. 40 LEDs were connected in parallel in the "SYSU" array (Figure S11b). 40 LEDs were lighted by the IMER driven using EMET of 20 Hz rotating magnetic field (Figure S11c-d). All the LEDs shone out brightly and uniformly.

12. Design of EMET with multiple N/S poles

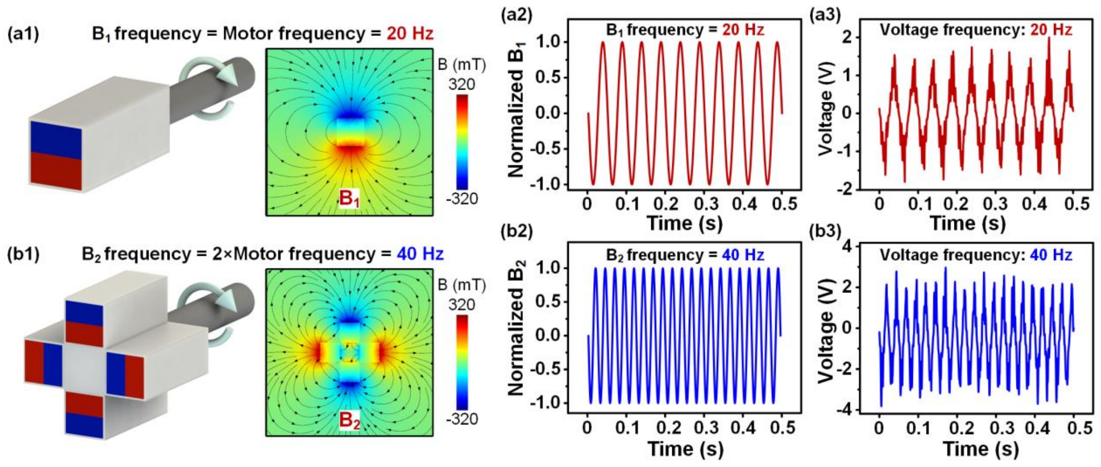

Figure S12. The magnetic field distribution and the rotating field frequency of the EMET with multiple N/S poles, and the output voltage of IMER driven by the transmitters.

The output power significantly increased with magnetic field frequency, which also can be easily adjusted via the magnetic poles of the transmitter. When there was single pair of N and S poles, the magnetic field produced single cycle of alternating current for single rotation of the motor. The frequency of the magnetic field kept same as the rotation frequency of the drive motor (Figure S12a1). When the driving motor rotated at frequency of 20 Hz, IMER could generate V_{oc} with frequency of 20 Hz and average amplitude of 1.6 V (Figure S12a2-a3). When there was two pairs of N and S poles, the magnetic field produced two cycles of alternating current for single rotation of the motor. The frequency of the magnetic field was twice the rotation frequency of the drive motor (Figure S12b1). When the driving motor rotated at frequency of 20 Hz, IMER could generate V_{oc} with frequency of 40 Hz and average amplitude of 3 V (Figure S12b2-b3). It can be seen that by increasing the magnetic poles of the magnet, the frequency of IMER signals can be increased without changing the frequency of the motor, and the electric performance can be significantly improved.

13. The current outputs of IMER under different conditions

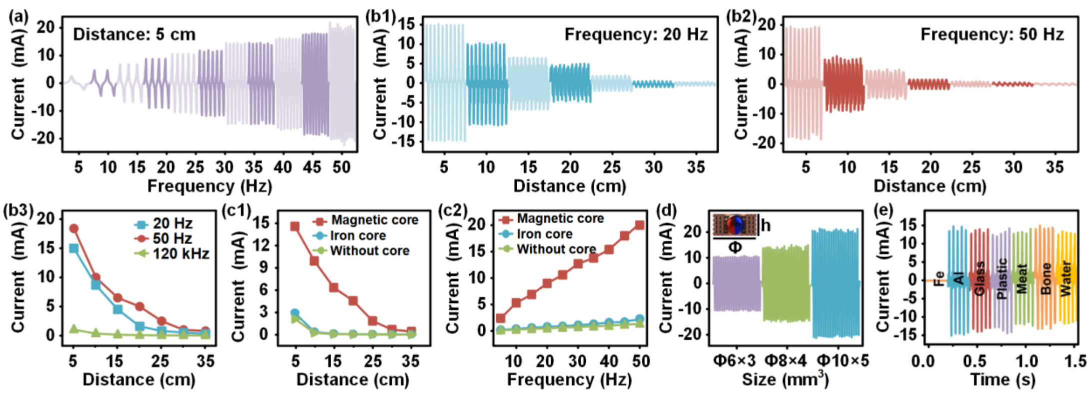

Figure S13. **(a)** Output current of the IMER under different magnetic field frequencies. **(b1-b3)** Output current of the IMER under different transmission distances. **(c1-c2)** Output current of the IMER

assembled with magnetic core, iron core, and without core under different transmission distances and magnetic field frequencies. **(d)** The output current of IMER with the size of Φ 6×3 mm^3, Φ 8×4 mm^3, and Φ 10×5 mm^3. **(e)** Current outputs of the IMER shielded by typical materials.

The electric output performance of IMER under different conditions, including magnetic field frequencies, transmission distances, IMER sizes and shielding materials, were further investigated. The output electric waveform upon the increase of magnetic field rotating frequency was shown in Figure S13a. The results found that increase of magnetic field rotating frequency significantly improved the electric output Isc. The Isc increased from 2.32 mA to 20.34 mA with the frequency increased from 5 Hz to 50 Hz, while the IMER could effectively output electricity of 2.6 mA even at an ultra-low rotating frequency of 5 Hz. The electric output waveforms (Figure S13b1-b2) and statistical performance (Figure S13b3) of IMER at different transmission distance between IMER and the EMET were then evaluated under typical rotating frequencies of 20 Hz and 50 Hz, while the electric outputs by high-frequency electromagnetic radiation at 120 kHz was employed for comparison. At 20 Hz ULFMEF operation, the electric outputs of IMER exhibited a slow attenuation from 15 mA to 5 mA upon increase of the transmission distance from 5 to 20 cm, and rapidly attenuated to 0.8 mA when the transmission distance increased to 35 cm. This was mainly because the EMET could effectively drive the rotating of MIFC to generate remarkable electric output (5 mA) for a long distance of ~20 cm, but the rotation of MIFC became significantly compromised for distance >25 cm. At 50 Hz ULFMEF operation, the electric outputs of IMER displayed a reduction from 18.4 mA to 8.7 mA upon when distance increased from 5 to 15 cm, but the electric output could maintain in a considerable level of ~1.6 mA even when the distance was increased to 20 cm. In contrast, the high-frequency electromagnetic radiation at 120 kHz showed a rapid attenuation from 0.98 mA to 0.02 mA when distance >5 cm, indicating the incapability of high-frequency electromagnetic radiation to transmit energy for longer distance. The essential role of the MIFC in ULFMEF was also investigated, where the IMER was designed without MIFC or with an embedded iron core instead of MIFC as control groups (Figure S13c). In these control groups, the energy transmission was mainly through the conventional pathway of electromagnetic induction via external magnetic field. The IMER without MIFC or with an embedded iron core instead of MIFC both displayed attenuated electric waveforms with I_{sc} of ~3 mA, which were ~5-folds lower than the MIFC-IMER. Moreover, the electric outputs of IMER were obviously improved for ~54-folds under transmission distance of 15 cm (Figure S13c1) and ~11-folds under magnetic field frequency of 50 Hz (Figure S13c2) due to the existence of MIFC compared to the control groups, demonstrating the MIFC assembled in energy receiver can significantly enhance the power generation performance. Because the MIFC in the coil provided an in-situ dynamic rotating magnetic field without attenuation to induce electric potential in IMER, thereby improving the electric output performance, weakening the field attenuation, and enhancing the transmission distance. According to the different diameters of MIFC (3 mm, 4 mm, 5 mm), the electricity performance of IMER in three sizes including Φ 6×3 mm^3, Φ 8×4 mm^3, Φ 5×10 mm^3 were measured (Figure S13d). The Φ 6×3 mm^3 IMER produced I_{sc} of 10 mA, the Φ 8×4 mm^3 IMER produced I_{sc} of 15 mA, and the Φ 10×5 mm^3 IMER generated I_{sc} of 20 mA. The results showed that the electric output performance of the IMER dramatically increased with the size. The detailed parameters and electric output performance were listed in Table S1. The electric output performances of the IMER were measured in enclosed space shielded with different materials. Figure S13e showed the I_{sc} waveforms of the IMER shielded by iron, aluminum, glass, plastic, meat, bone and water closed boxes. The electrical outputs of IMER shielded by iron box decreased significantly, only producing I_{sc} of 10 μA, due to the high magnetic permeability of iron, which can gather the magnetic line along the box, weakening the

magnetic interaction on the MIFC of IMER. However, the output voltage could be maintained in range of ~12 mA-14.8 mA. The results showed that the shielding effect of aluminum, glass, plastic, meat, bone and water on the electrical output of IMER can be ignored. The ULFMEF system exhibited high magnetic field penetration in high conductivity and low magnetic permeability materials (such as aluminum).

14. Electric signals of the IMER under 10-100 Hz magnetic fields

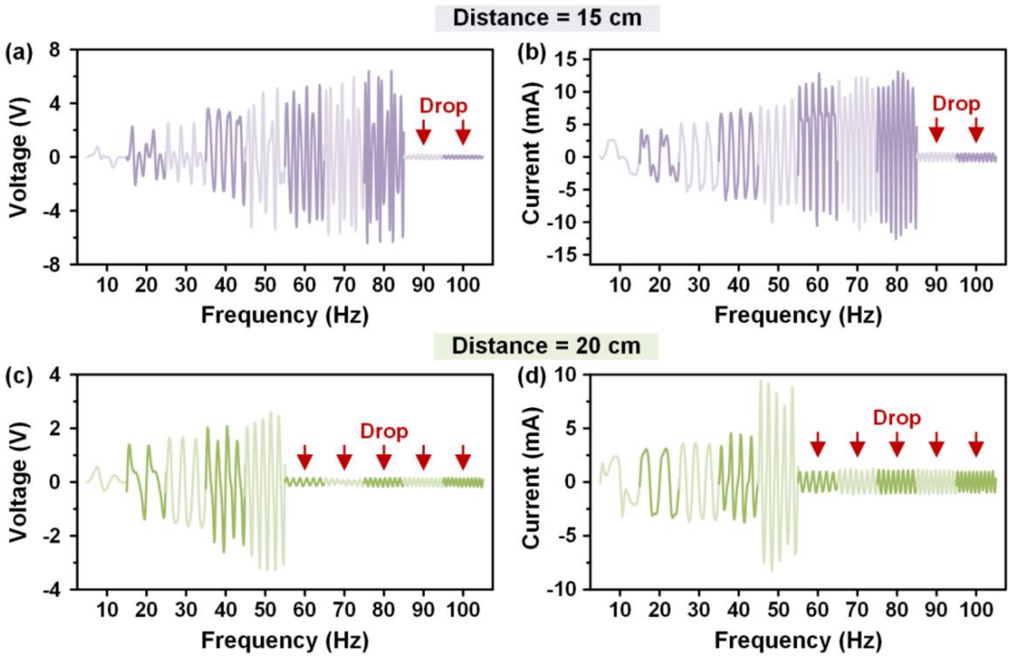

Figure S14. Measured electric signals of the IMER under 10-100 Hz magnetic fields at **(a)** 15 cm and **(b)** 20 cm from the external transmitter.

In theory, the larger the rotational frequency of the magnetic field, the higher the output voltage. In order to determine the optimal frequency, the rotational frequency of the magnetic field was increased from 10 Hz to 100 Hz and the electrical output signals of the IMER was measured at 15 cm and 20 cm from the EMET. When the transmission distance was 15 cm, the output electric signals increased when the rotational frequency changed from 10 Hz to 80 Hz. As the magnetic field frequency continued to increase to 90 and 100 Hz, the electrical output dropped significantly (Figure S14a-b). When the transmission distance was 20 cm, the output electric signals increased when the rotational frequency changed from 10 Hz to 50 Hz. And the electrical outputs went downward when the magnetic field frequency continued to increase >50 Hz (Figure S14c-d). The results showed that the electric performance could not continuously increase with the magnetic field frequency. This may be due to the fact that the friction between the magnetic ball and the coil increased with the rotation frequency. The increased friction resulted in non-synchronous rotation of the magnetic ball, causing a decrease in output power. According to the experimental results, when the transmission distance was 15 cm, the optimal frequency was 80 Hz. And when the transmission distance increased to 20 cm, the optimal frequency decreased to 50 Hz. Therefore, 50 Hz was set as the maximum magnetic field frequency used in this paper.

15. FFT of the signals under different frequency

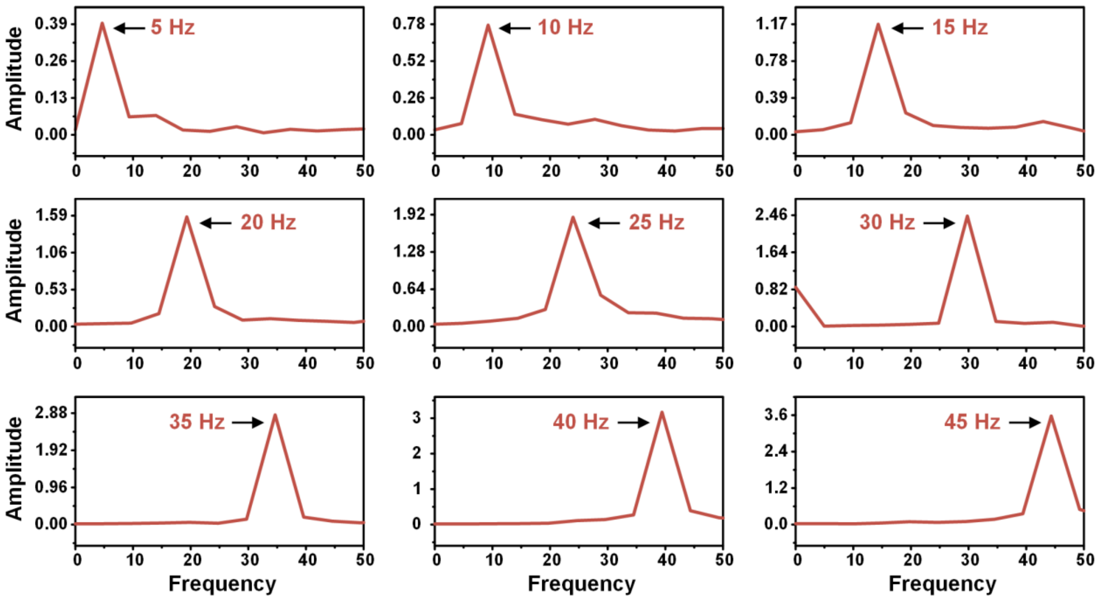

Figure S15. FFT of the electric output signals of IMER driven by different field frequencies.

The electric output signals of IMER driven by different field frequencies were Fast Fourier transformed and the frequency distribution was obtained. The transformed characteristic peaks were well consistent with the rotating field frequency, demonstrating the rotation behaviors of MIFC in IMER and the driving magnet were synchronous.

16. Electrical outputs of IMER under high-frequency electromagnetic field

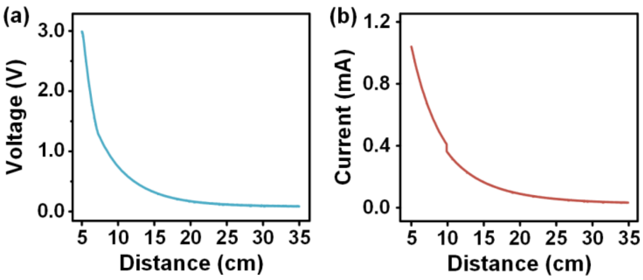

Figure S16. **(a)** The output V_{oc} and **(b)** I_{sc} of IMER under the high-frequency electromagnetic field.

The electrical output performance of the IMER under a high-frequency electromagnetic field (120 kHz) was measured. The IMER could output Voc of ~3 V and I_{sc} of ~0.95 mA close to the high-frequency magnetic field source. While the output electricity of IMER decreased rapidly in an exponential tendency when the IMER was gradually moved away from the high-frequency magnetic field source.

17. Time and frequency domain signals under different transmission distances

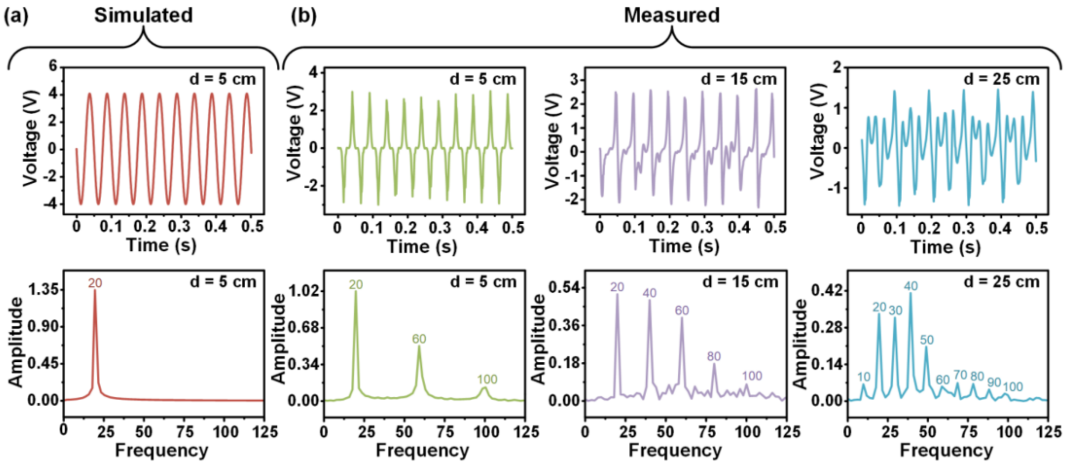

Figure S17. The time and frequency domain signals of IMER under different transmission distances.

When the transmission distance increased, the signal distortion and unexpected fluctuation of electric output could be found in time-domain plots. The output electrical signals of IMER in the time domain driven by 20 Hz rotating magnetic field were Fast Fourier transformed and the frequency distribution was obtained (Figure S17). It can be found that the unexpected characteristic peaks increased with transmission distances. The possible reason is that the magnetic interaction between the MIFC and driving magnet gradually decreased with the transmission distance, resulting in the step-missing, oscillating or crawling behaviors of the MIFC during the rotation.

18. Long-term stability of ULFMEF.

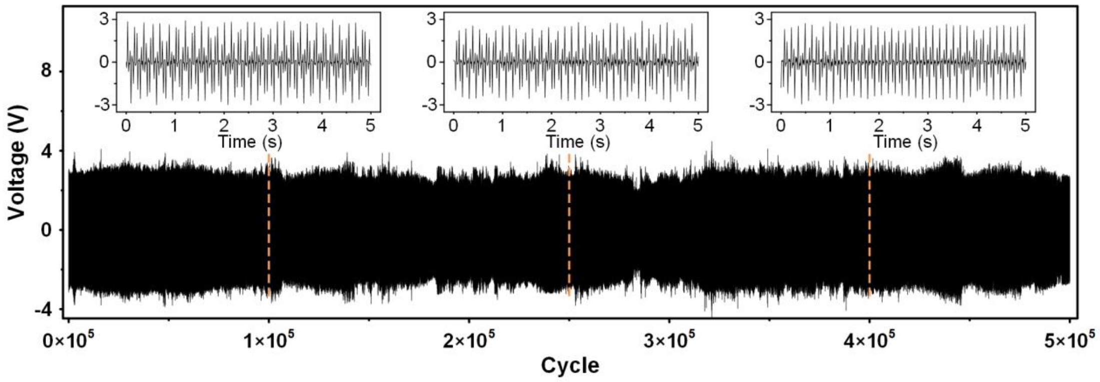

Figure S18a. Measured output voltages of the ULFMEF system over 500,000 cycles lasted for about 6.94 h.

The stability and durability of the ULFMEF were tested over 500,000 cycles as shown in Figure S18. The results suggested that the IMER could stably output voltage waves with a peak of ~3.1 V even for 6.94 hours of rotation, showing good mechanical and electrical reliability.

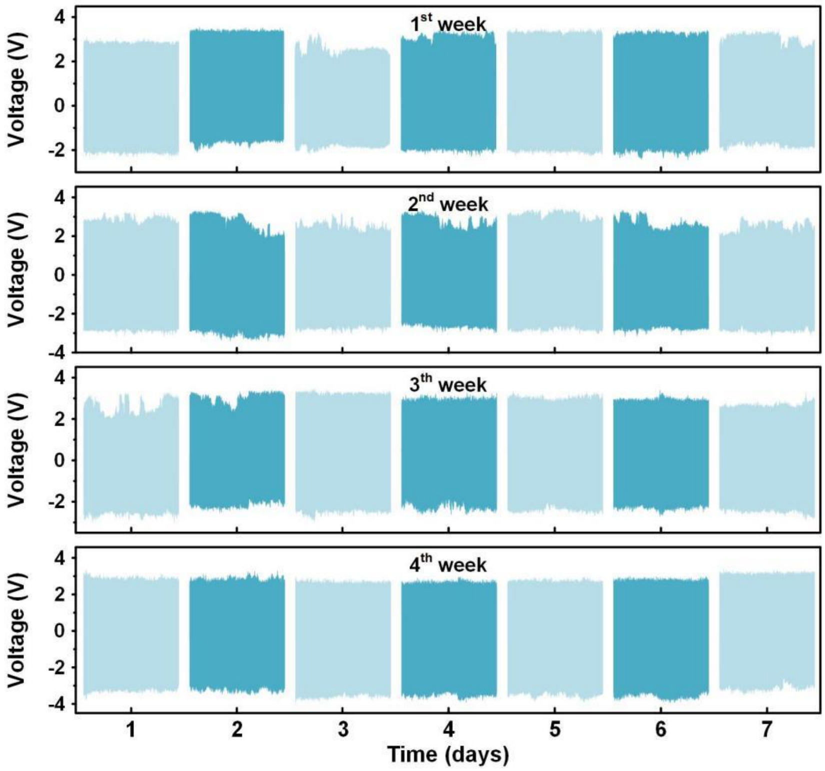

Figure S18b. Measured output voltages of the ULFMEF system during four weeks.

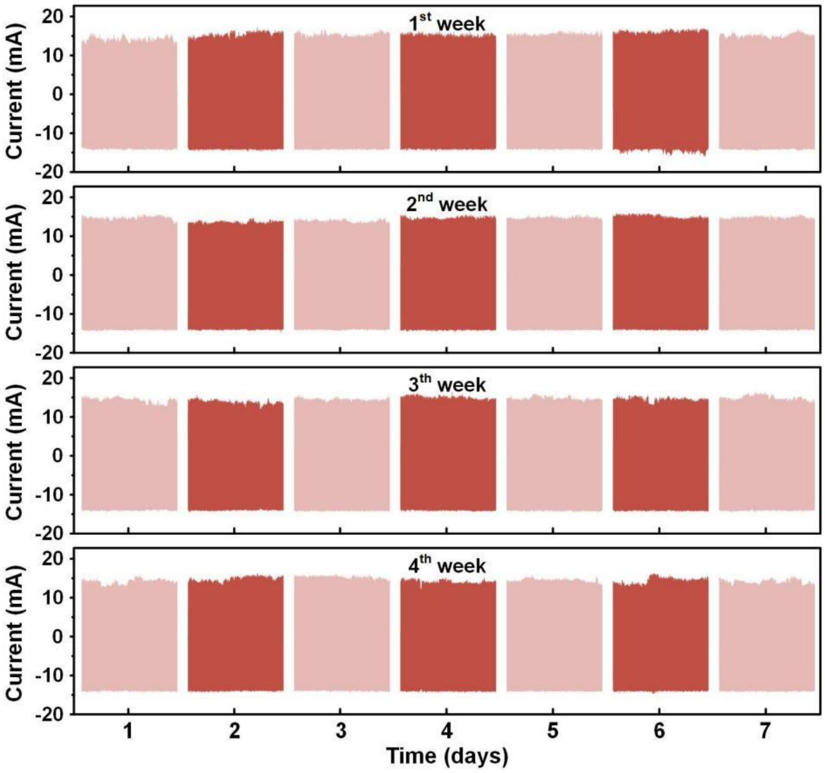

Figure S18c. Measured output currents of the ULFMEF system during four weeks.

To further investigate the long-term stability, the voltage and current outputs of the IMER in the ULFMEF system were measured during one month. The ULFMEF system consistently generated stable electrical signal outputs each day, with voltage peaks fluctuating within a range of -3 V to 3 V

(Figure S18b), and current peaks varying within a range of -15 mA to 15 mA (Figure S18c). The results indicated that the ULFMEF system exhibited long-term stability in its electrical performance.

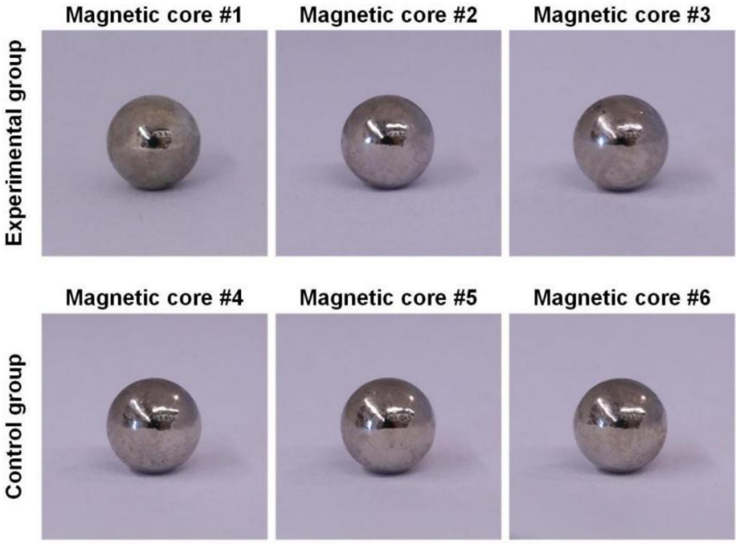

Figure S18d. Surface morphology of the magnetic balls in the experimental group and control group, respectively.

After one month of continuous operation, the IMER was disassembled into the internal magnetic ball and external copper coil. For the magnetic ball, the surface morphology was observed in Figure S18d and magnetic property was tested. Optical images revealed that the surface of the magnetic balls showed no signs of damage, maintaining their structural integrity during continuous rotation for one month.

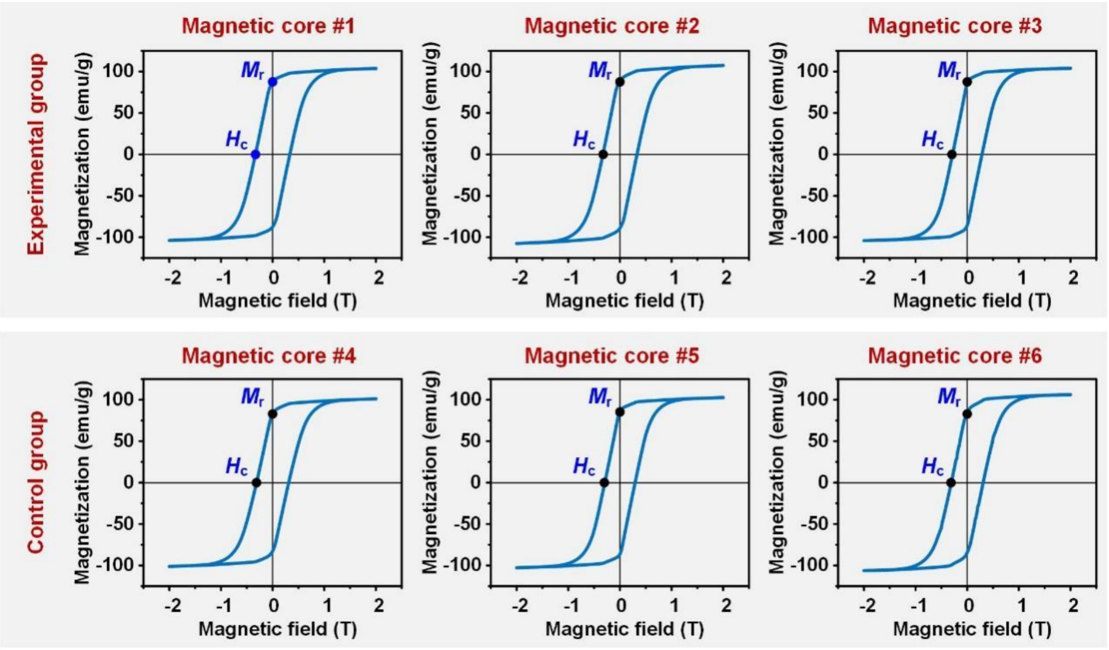

Figure S18e. Magnetic hysteresis loop of the magnetic balls in the experimental group and control group, respectively.

The magnetic hysteresis loops of the magnetic balls were measured as shown in Figure S18e. The residual magnetization Mr of the magnetic balls of the experimental group was in the range of 85.64–88.24 emu/g, which was similar to that of the control group in the range of 83.28–86.73 emu/g. The

coercivity H_c of the magnetic balls of the experimental group was in the range of 299–326 mT, which was similar to that of the control group in the range of 302–314 mT. The results showed the excellent magnetic properties of the magnetic balls after continuous rotation for 1 month.

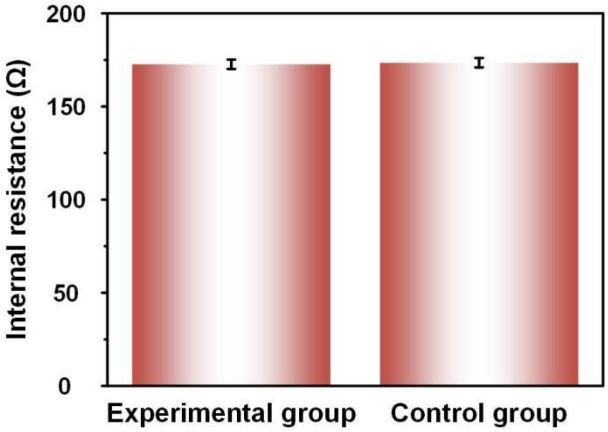

Figure S18f. Internal resistances of the copper coils in the experimental group and control group, respectively.

As shown in Figure S18f, the measured internal resistance of the coils in both the experimental and control groups were approximately 172.6 Ω and 173.5 Ω respectively. There was no significant difference between two groups, indicating the structural integrity of the coils.

19. Temperature change of the working IMER

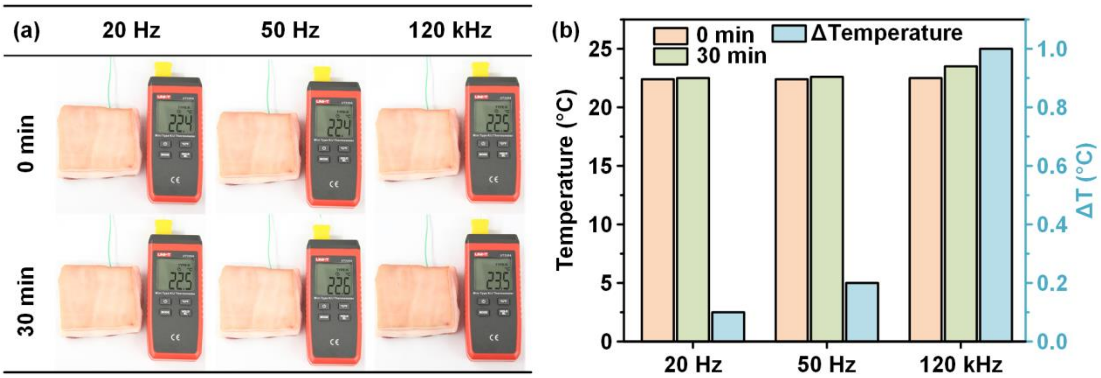

Figure S19. **(a)** The working IMER buried in tissue under 20 Hz, 50 Hz and 102 kHz magnetic field operation. **(b)** Measured internal temperature changes of the tissue.

To investigate the generated heat, the internal temperatures of the working IMER buried in tissue were measured by the thermocouple thermometer (UT320A, UNI-T, China) under 20 Hz, 50 Hz magnetic field, respectively. As control, the tissue temperature changes under high-frequency electromagnetic radiation at 120 kHz was employed for comparison. As shown in Figure S19a, the internal temperatures of the pork tissue were recorded during the ULFMEF operation at 0 min and 30 min. Under 20 Hz magnetic field, the internal temperature changed from 22.4 ℃ to 22.5 ℃ with 0.1 ℃ increment. Under 50 Hz magnetic field, the internal temperature changed from 22.4 ℃ to 22.6 ℃ with 0.2 ℃ increment. While under 120 kHz high-frequency electromagnetic field, the internal temperature changed from 22.5 ℃ to 23.5 ℃ with 1.0 ℃ increment (Figure S19b). According to the experimental

results, it can be seen that IMER produced little heat when operated under a 20 Hz magnetic field in the pork tissue, while there was a large temperature increase under 120 kHz magnetic field.

20. Relative angles and positions between the rat and EMET in vivo test

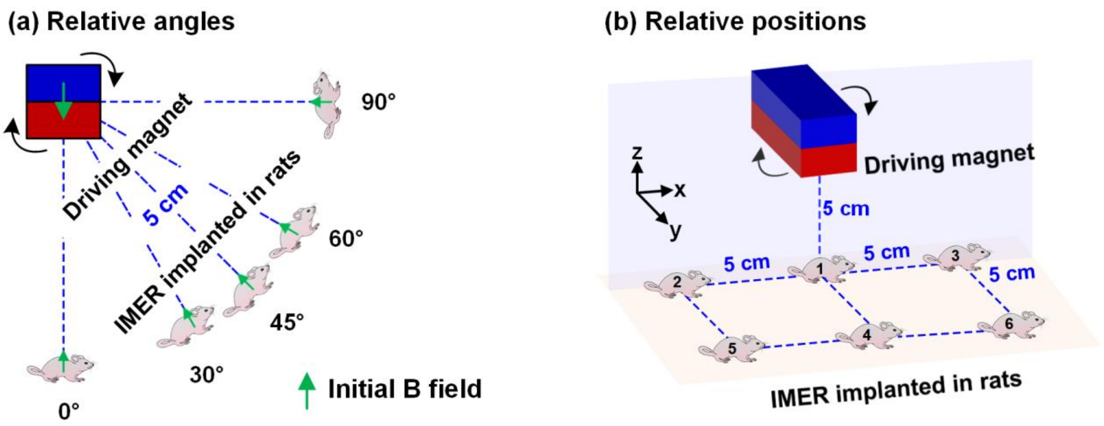

Figure S20. **(a)** Illustration of the relative angles and **(b)** positions between the EMET and IMER implanted in rats.

The rats were deeply anesthetized with 2% isoflurane. After being deeply anesthetized, the hair on the rat's back was completely removed, and then a small skin incision was made on the dorsal. The skin was separated from the underlying fascia using a needle clamp, and the IMER was then implanted into the subcutaneous pocket opened by the blunt dissection. The incision was carefully stitched with 4-0 surgical sutures. The rats implanted with IMER were placed under the EMET. The output electricity performances of IMER implanted in the rat were measured using the electrometer under the rotating magnetic field with different angles and positions. The output voltages of IMER at different relative angles (Figure 4i3) and positions (Figure 4i4) were measured at angle 0 °~ 90 °and position 1 ~ 6. The relative angles and positions between the EMET and IMER were defined and illustrated in Figure S20. The working angles were 0 °, 30 °, 45 °, 60 ° and 90 ° and the transmission distance was 5 cm (Figure S20a). The position 1 to 6 were located in the same plane (X-Y plane), and the distance between the adjacent positions was 5 cm (Figure S20b). Position 1 was located directly below the transmitter with a vertical distance of 5 cm.

21. Design of the battery-free optoelectronic stimulator

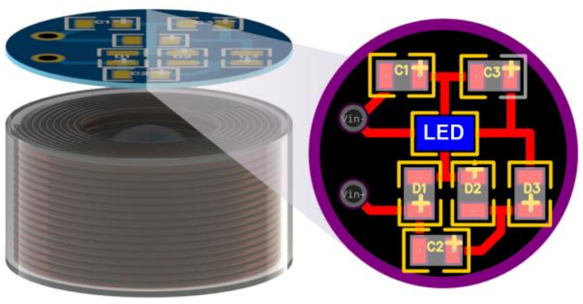

Figure S21. Design of the wireless and battery-free optoelectronic stimulator.

A wireless and battery-free optoelectronic stimulator based on IMER was designed for

optogenetic brain neuromodulation (Figure S21). The optoelectronic stimulator was composed of the IMER, the voltage multiplier circuit, and the blue μ-LED. The Φ 6×3 mm^3 IMER was selected for optoelectronic stimulator. The blue μ-LED was lightened via the IMER and the voltage multiplier circuit driven by the external rotating magnetic field.

22. The power attenuation of the ULFMEF technique through bone tissue

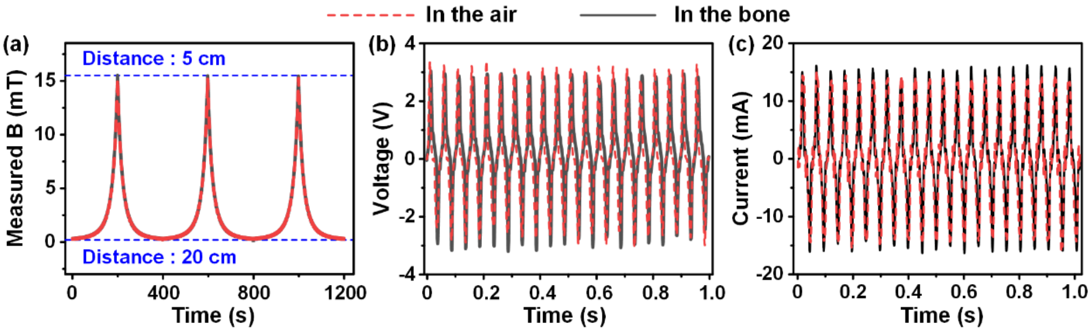

Figure S22. (a) Measured magnetic flux densities of the EMET when the probe is placed in air and in bone, respectively. **(b-c)** Measured output voltages and currents of the IMER in air and in bone, respectively.

The power transfer and attenuation of the ultra-low frequency magnetic energy focusing technique were investigated through bone tissue in two ways. Firstly, the magnetic field strength of the EMET was tested through bone tissue. The Gauss meter probe was placed in air and in a pig leg bone, respectively, and the distance between the EMET and the probe varied from 5 cm to 20 cm. The probe recorded the flux density waveform of the EMET over three cycles, as shown in Figure S22a. When the distance between the probe and the EMET was 5 cm, the detected magnetic flux densities in air and in bone tissue were 15.52 mT and 15.50 mT, respectively. When the distance was increased to 20 cm, the magnetic flux densities in air and bone tissue were 0.33 mT and 0.34 mT, respectively, which did not show large differences. In addition, the IMER was placed in the air and bone tissue, of which the electric performances were measured, respectively. The open-circuit voltage and short-circuit current waveforms of the IMER were shown in Figure S22b-c. In the air, the IMER could generate peak to peak voltage (V_{pp}) of 5.81 V, and peak to peak current (I_{pp}) of 27.6 mA. In the bone tissue, the IMER could generate V_{pp} of 5.96 V, and I_{pp} of 29.1 mA. The results showed no obvious differences in the electric performances of the IMER in air and bone tissue. The above experimental results showed that the bone tissue didn't absorb low frequency magnetic field, which may not induce the attenuation on the electric performance of the ULFMEF system.

23. Luminescence power of wireless optogenetic stimulation devices

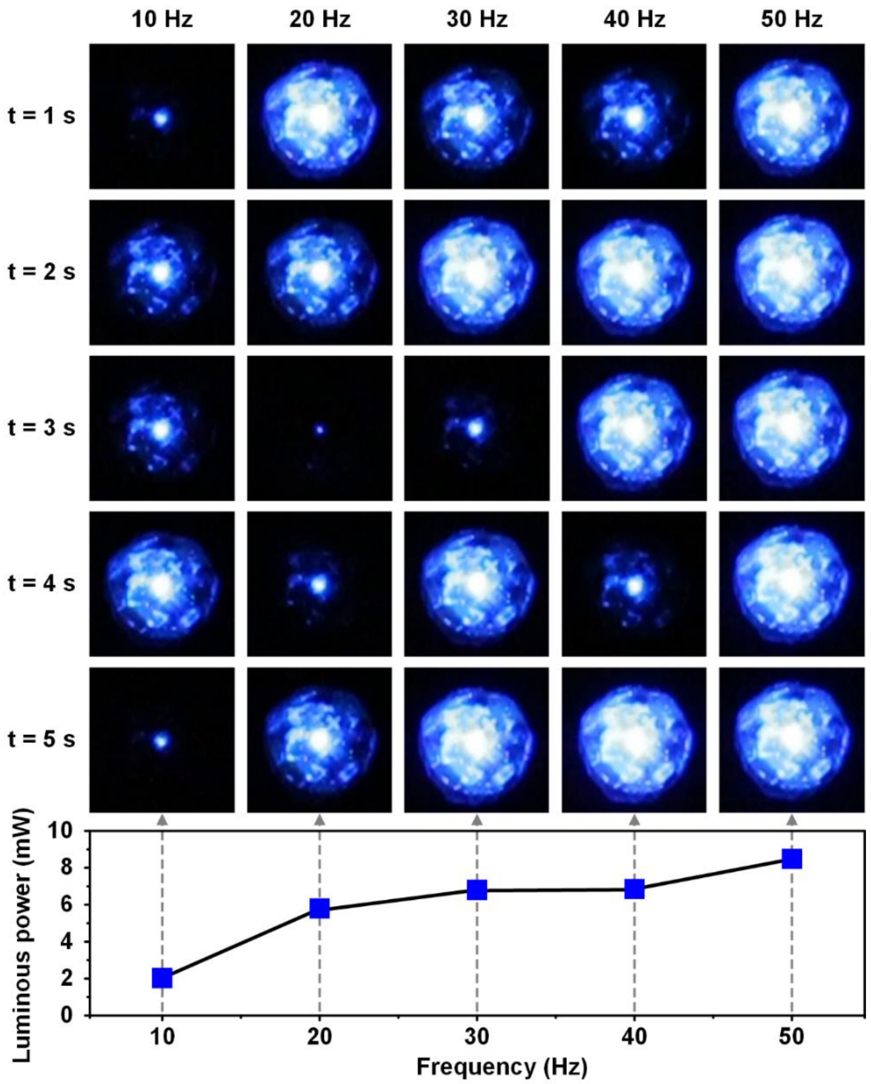

Figure S23a. Luminous intensities of the IMER-driven LED at different magnetic field frequencies from 10 Hz to 50 Hz.

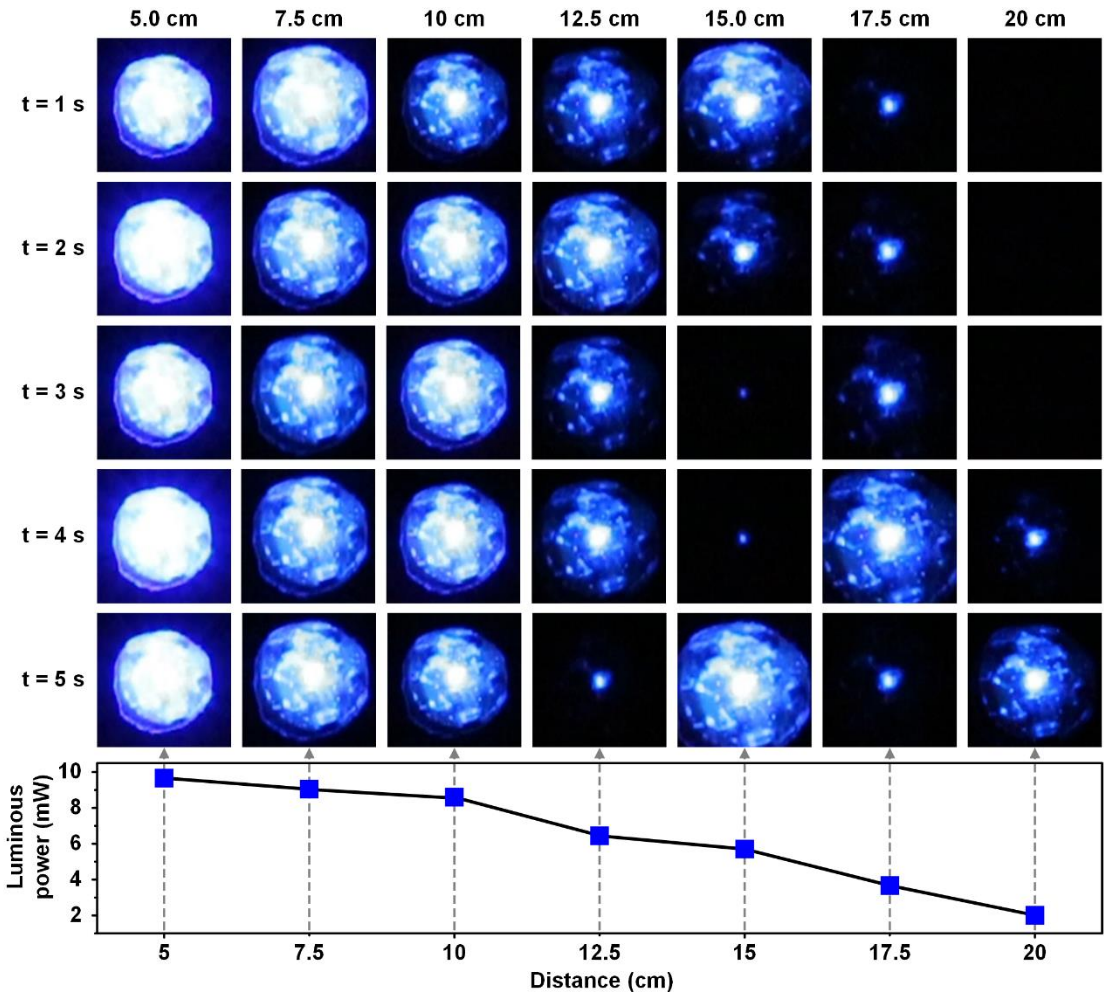

Figure S23b. Luminous intensities of the IMER-driven LED at different transmission distances from 5 cm to 20 cm.

The luminous intensity of the implanted device depended on the output power of ULFMEF, while the power can be adjusted by changing the frequency and transmission distance of the driving magnetic field. When the transmission distance of the IMER and the EMET was 5 cm, the average luminous intensities of the IMER-driven LED from 1 s to 5 s were measured under different magnetic field frequency (10 Hz to 50 Hz). According to the recorded series of images, it could be seen that the LED showed weak brightness with luminescence power of ~2 mW when the rotational frequency of the EMET was 10 Hz. When the magnetic field frequency increased, the brightness of the LED gradually increased. The brightness of the LED got maximized with luminous power of ~8.5 mW when the magnetic field frequency was 50 Hz. This is because the electric performance of the IMER increased with the magnetic field frequency, promoting the luminous power of the IMER-driven LED.

Additionally, when the external magnetic field frequency was 50 Hz, the average luminous intensities of the IMER-driven LED from 1 s to 5 s were measured under different transmission distances (5 cm to 20 cm). When the transmission distance was 5 cm, the brightness of the LED was maximized with luminous power of about 9.46 mW. While when the transmission distance increased, the brightness of the LED got diminished. The brightness of the LED got minimized with luminous power of ~2 mW until the transmission distance was 20 cm. This is because the magnetic energy received by the IMER decreased with the increasing transmission distance, resulting in reduced electric

performance and luminous power. However, the IMER-driven LED could still provide 6.44 mW luminous power up to 12.5 cm, which is enough for optogenetic stimulation.

24. Experimental setup for the optoelectronic stimulation

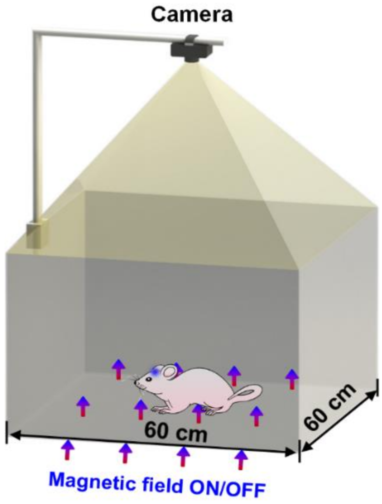

Figure S24. Experimental setup for the behavior analysis of optogenetic rat implanted with optoelectronic stimulator under rotating magnetic field.

The rats were anesthetized with 1% pentobarbital sodium solution by intraperitoneal injection. The deeply anesthetized rats were fixed to a stereo locator. An incision was made at the midline of the scalp to expose the skull. Then another incision (diameter: 1 mm) was created on the skull surface positioned at AP: 2.2, ML: 1.7 mm using a cranial drill. The capillary injection tube was positioned at M1 brain region (AP: 2.2, ML: 1.7, DV: -2.2 mm) of the motor cortex using a stereoscopic locator. Then 1.5 μL optogenetic virus AAV-CaMKIIa-ChR2-mCherry was injected into the target using a microinjection pump at a rate of 0.3 μL/min. After the injection, the needle was left in place for ten minutes and then slowly pulled out.

An incision (diameter: 1.5 mm) was opened on the skull surface (AP: 2.2, ML: 1.7 mm) using a cranial drill. The light-guiding post of the photo-stimulation device was fixed into the craniofacial incision and implanted in the brain motor cortex. The other three incisions (diameter: 0.5 mm) were drilled on the cranial surface around the implantation site, and the corresponding cranial nails (1 mm × 3 mm) were inserted. The dental cement (I, Zhuhaokang, Shanghai) was used to fix the light-stimulation device and cranial nails, and then cover the craniofacial surface.

For the open field behavior test, a square activity room (60×60 cm2) was built without obstacles, allowing the rats to move freely. The EMET was operated under the activity room to provide a low-frequency rotating magnetic field for optogenetic stimulation. The optogenetic stimulator could be activated only when the magnetic field was switched on. The motion behaviors and trajectories of the rats were recorded by the camera for 30 minutes and then analyzed using image analysis software (Image J, USA).

25. Multi-angle magnet array for long-distance magnetic energy transfer.

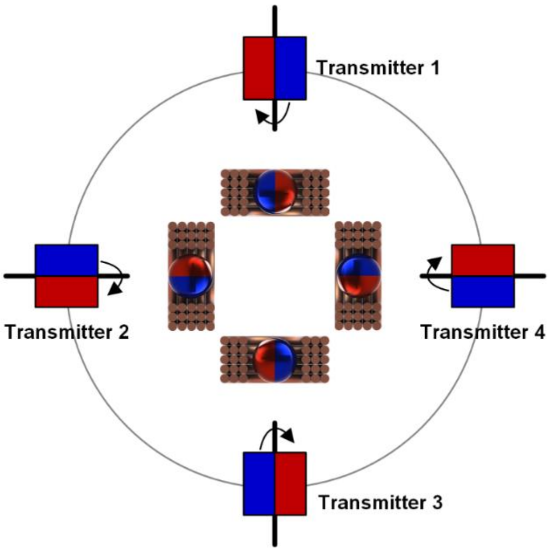

Figure S25. Multi-angle magnet array for long-distance magnetic energy transfer.

For practical applications, the multi-angle magnet array was designed to remotely power a battery-free implantable micro-LED buried in the head of moving rats in a cage, which can power battery-free implantable devices over long distance. The power generation performance of the IMER could be maintained at different angles and orientations within the range of the magnetic field array.

26. Design of the smartphone APP

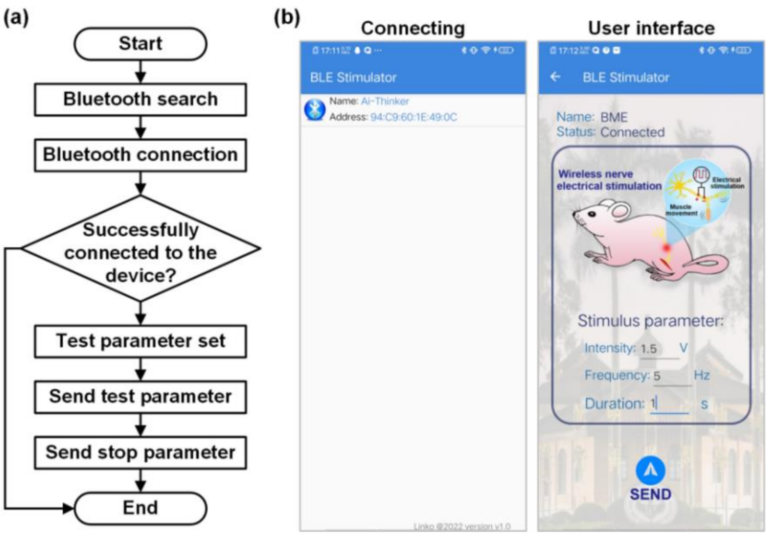

Figure S26. **(a)** Flow chart of the smartphone APP. **(b)** Connection between the APP and nerve bioelectronic stimulator via Bluetooth module, and the user interface of smartphone APP.

The flow chart of the smartphone APP was shown in Figure S26a. The smartphone APP firstly connected with the wireless nerve bioelectronic stimulator via Bluetooth (Figure S26b). Then smartphone APP sent the stimulation commands and parameters (including stimulation intensity,

frequency, and duration) to the wireless nerve bioelectronic stimulator.

27. Circuit design of the wireless nerve bioelectronic stimulator

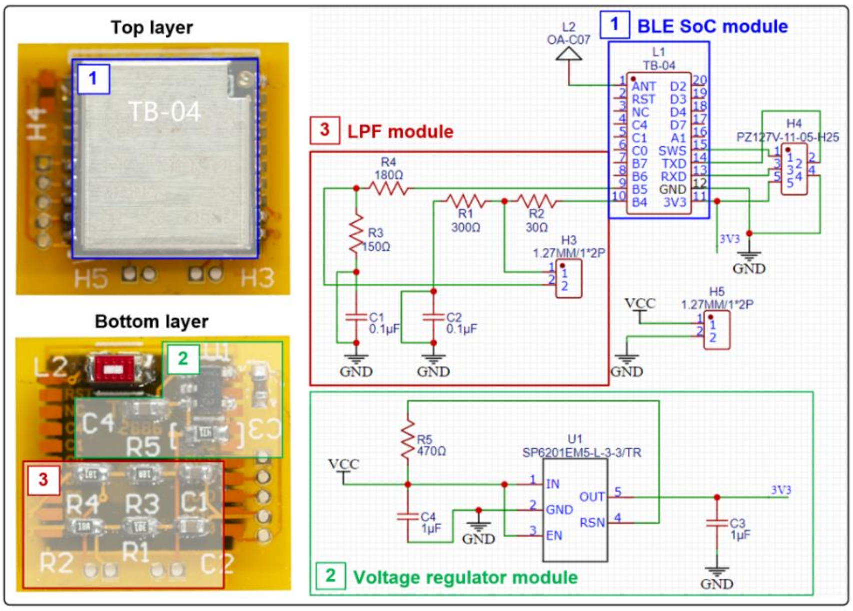

Figure S27. The circuit design of the wireless nerve bioelectronic stimulator.

The circuit design of the wireless nerve bioelectronic stimulator based on the IMER was presented in Figure S27. The chip TLSR8250F512ES16 was selected as the microcontroller unit (MCU) of the TB-04 module. The Bluetooth of the TB-04 module could realize wireless data communication in low energy waste. The rechargeable battery was charged via the IMER. The output voltage for the TB-04 module was tuned via the voltage regulator module. The voltage regulator module utilized a linear regulator SP6201EM5-L-3-3/TR combined with decoupling capacitors, which can transform the 3.6 V input to a stable 3.3 V output. The adjustable analog voltage for nerve stimulation was D/A converted via the PWM output of MCU combined with the RC low-pass filter. The produced potential difference between the cathode and anode (at H3 port) can be calculated as follows:

$V_{\text{out}} = V_{+} - V_{-}$ (S5)

where the $V_{+} = 0 \sim \frac{R_1}{R_1+R_2} \times 3.3$ V, $V_{-} = \frac{R_3}{R_3+R_4} \times 3.3$ V, the values of V_{+} and V_{-} are determined by the resistance of the low-pass filter circuit. The cathode and anode ports could generate adjustable voltage by selecting different resistance, so the electric outputs were designed as follows:

As the R1, R2, R3 and R4 were set as 100 Ω, 230 Ω, 50 Ω and 280 Ω, respectively. The output voltage for stimulation was:

$V_{+} = 0 \sim 1$ V, $V_{-} = 0.5$ V, $V_{\text{out}} = -0.5 \sim 0.5$ V (S6)

As the R1, R2, R3 and R4 were set as 200 Ω, 130 Ω, 100 Ω and 230 Ω, respectively. The output voltage for stimulation:

$V_{+} = 0 \sim 2$ V, $V_{-} = 1$ V, $V_{\text{out}} = -1 \sim 1$ V (S7)

As the R1, R2, R3 and R4 were set as 300 Ω, 30 Ω, 150 Ω and 180 Ω, respectively. The output voltage

for stimulation:

$V_{+} = 0 \sim 3$ V, V_{-}=1.5 V, V_{out} =-1.5 ~ 1.5 V (S8)

The actual voltages for stimulation were shown in Figure S30a.

28. Design of the flexible electrode sheet

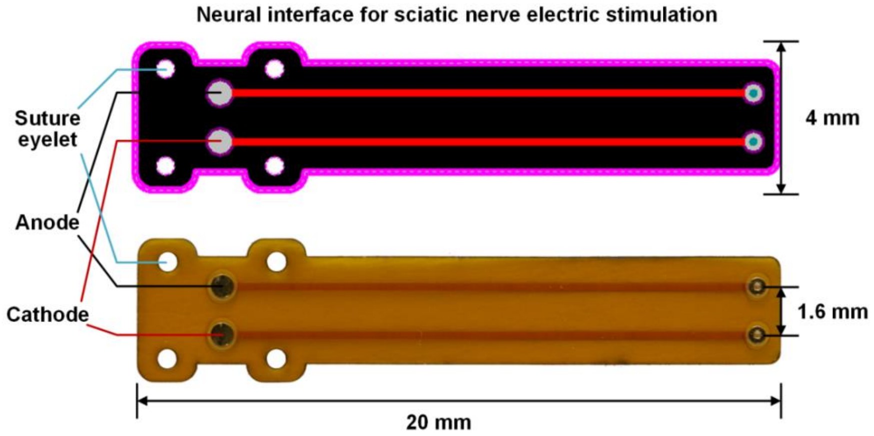

Figure S28. Design of the flexible electrode sheet for sciatic nerve stimulation.

A flexible electrode sheet was designed for sciatic nerve stimulation. The length and width of the flexible electrode sheet were 20 mm and 4 mm, respectively. The electrode sheet is made up of an external polyimide package and an internal copper electrode. The impedance of the electrode is approximately 1.4 Ω. The bare cathode and anode were coated with Au film, and the distance between two stimulus points was 1.6 mm. This flexible electrode sheets were manufactured by Shenzhen Star Extraordinary Technology Company. The flexible electrode sheet can tightly wrap the sciatic nerve, so a stable interface between the nerve and electrodes (anode and cathode) could be established.

29. *In vivo* smartphone-controlled nerve stimulation

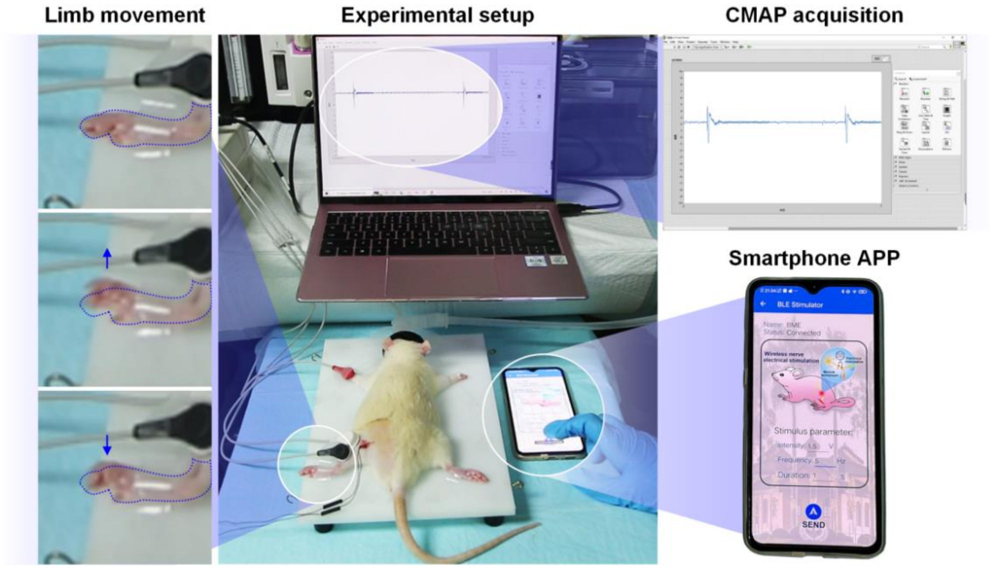

Figure S29. Experimental setup of smartphone-controlled nerve bioelectronic stimulation.

The rats were deeply anesthetized with 2% isoflurane. The hair on the hindlimb was removed, and a small skin incision was then made over the greater trochanter of the femur on the right flank. A blunt incision was made in the fascia connecting the gluteus maximus with the biceps femoris, and a plane was opened between the muscles, in which the sciatic nerve could be clearly accessible. The gluteus maximus was pulled caudally to expose the sciatic nerve, and the flexible electrode sheet was wrapped over the sciatic nerve. The sciatic nerve of the rat was stimulated by the output electricity of the electrodes from the IMER-based implantable neuromodulator, whose battery was wirelessly charged by the EMET. The smartphone APP could send stimulus signals to the electric stimulator via Bluetooth. The electric stimulator generated the corresponding stimulation voltages to excite the sciatic nerve. The electromyography electrodes were adhered to the skin surface of the hindlimbs and the action potentials of the complex muscle groups were collected during the stimulation using an electromyography instrument. Finally, the measured electromyography signals were collected by the data acquisition card and displayed on the computer via a Labview program.

30. Output voltages of the nerve electric stimulator

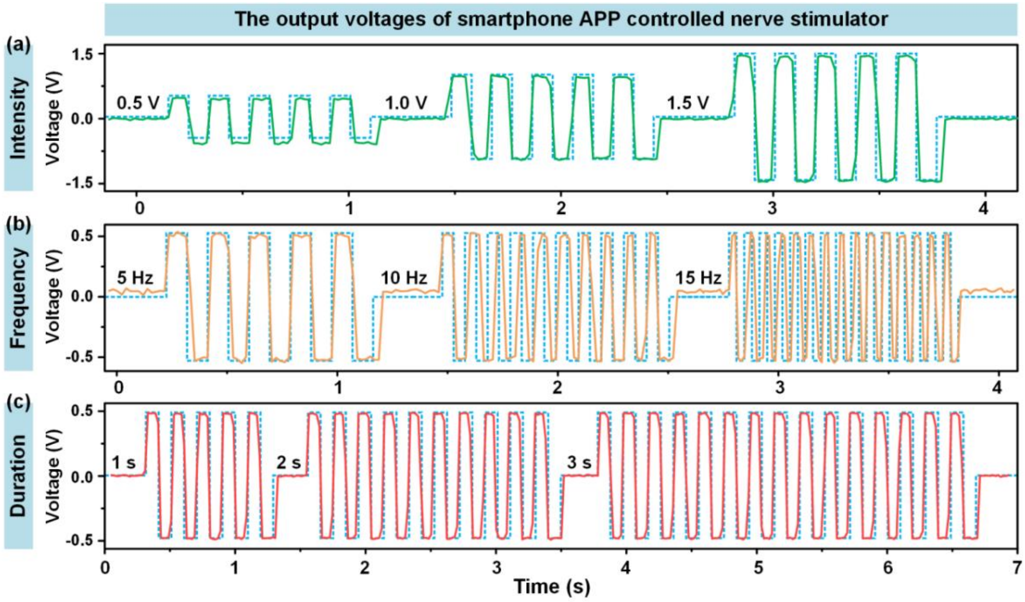

Figure S30. The output voltages of smartphone APP controlled nerve stimulator.

The output analog voltage for nerve stimulation could be adjusted via the selection of different resistance values. The designed (blue dot line) and measured (green solid line) voltage waveforms with the peaks of -0.5 ~ 0.5 V, -1 ~ 1 V and -1.5 ~ 1.5 V were shown in Figure S30a. These two lines are very close, demonstrating ideal output electricity for nerve bio-electronic stimulation. Besides, the output electricity frequency at 5 Hz, 10 Hz, and 15 Hz also could be designed for nerve stimulation by tuning the PWM pulse (Figure S30b). Furthermore, the duration of the output electricity for nerve stimulation also could be adjusted from 1s to 3s (Figure S30c).

31. Full-body-scale wireless magnetic energy transmission bedplate

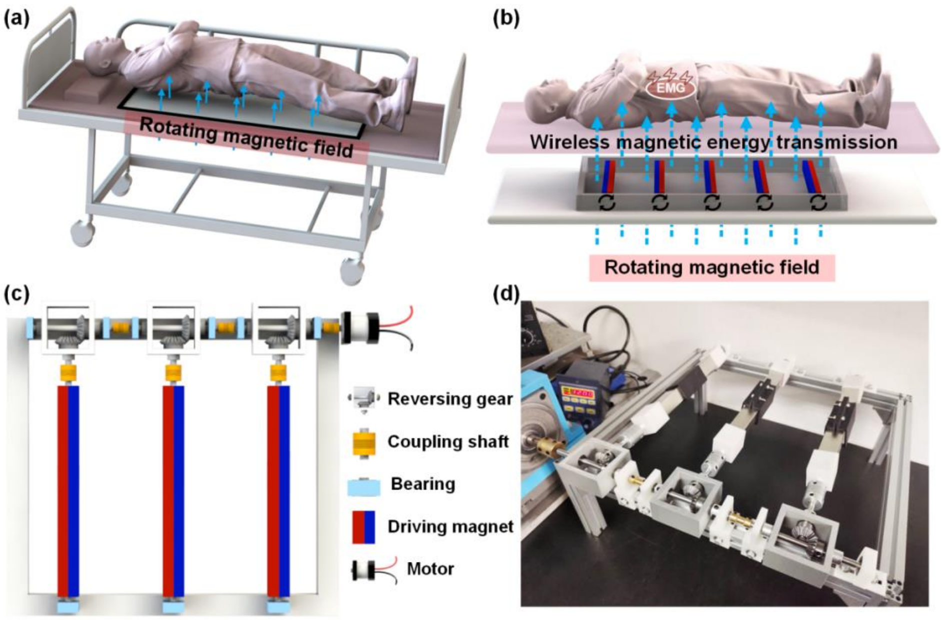

Figure S31. **(a)** Schematic illustration of the full-body-scale magnetic energy transmission system for wireless charging of the medical implants. **(b)** The rotating magnet array embedded in the bed for wireless energy transmission. (c-d) The pint-sized magnetic energy transmission system was designed and fabricated.

A full-body-scale magnetic energy transmission bedplate was designed for the wireless charging of the medical implants based on IMER (Figure S31a-b). The patient implanted with the medical electronic device could freely lay on the bedplate and then the devices could be wirelessly charged via the rotation of magnet array embedded in the bed. The magnetic energy transmission bedplate not only eliminated the potential risk in battery replacement surgeries but also provided high freedom for the patients. Based on this conception, a pint-sized external magnetic energy transmission system for wireless charging the medical electronic devices was designed and fabricated (Figure S31c-d). It mainly consisted of a driving motor, the commutation gears, the magnet array and several bearings. Driven by the motor, the magnet array was rotated to generate a time-varying magnetic field for medical implants charging.

32. Biosafety of the IMER

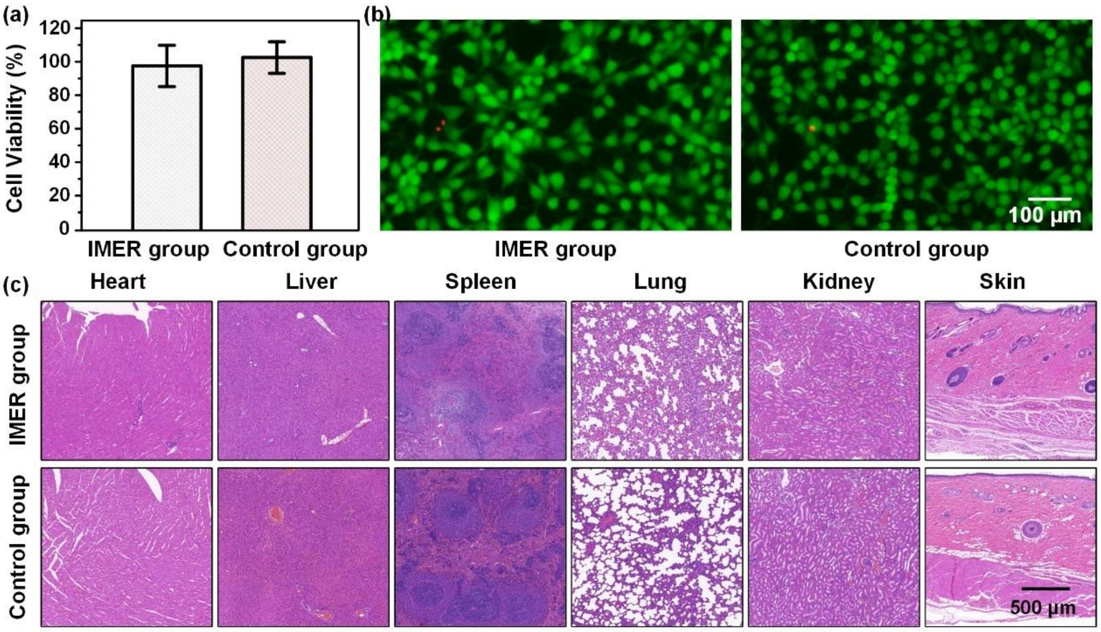

Figure S32. **(a)** Viability of 3T3 cells in IMER and control groups. **(b)** Fluorescence images of stained 3T3 cells in IMER and control groups. **(c)** H&E staining of vital organs and implant-position skin in IMER and control groups.

To confirm the biosafety of the ULFMEF system, the biocompatibility of the implanted IMER was tested using the MTT assay, Calcein AM/PI test and hematoxylin and eosin (H&E) staining, respectively. In MTT assay, the 3T3 cells were incubated in Dulbecco's modified Eagle's Medium (DMEM) supplemented with 10% (v/v) fetal bovine serum and 1% penicillin-streptomycin. The sterilized IMER was immersed in the original DMEM for 72 h to obtain a releasing media. The cell culture medium was then replaced by releasing culture media for another 24-h incubation. 250 μL Calcein AM/PI test compound was added and incubated in darkness for 30 min. The living and dead cells were stained with Calcein AM ($\lambda_{ex/em}$: 485/535 nm) and PI ($\lambda_{ex/em}$: 530/620 nm), respectively. The results showed that the relative viability of 3T3 cells in releasing media of IMER group was about 97.5 % (Figure S32a), showing healthy level of cell survival. In Calcein AM/PI test, the cells were then observed using a fluorescence microscope. The releasing media was removed and the cells were incubated for 4 h in the mixture of 20 μL MTT solution and 100 μL original culture media. The mixture was replaced by 150 μL dimethyl sulfoxide and shaken for 10 min. Finally, the absorbance was measured (wavelength: 570 nm) using a microplate reader. The morphologies of cells showed no obvious diminish of living cells (Figure S32b). In H&E staining, the rats implanted with the magnetic-core IMER for one month were dissected and their vital organs including heart, liver, spleen, lungs, kidneys, and skin at the surgical site were removed and pathologically observed. The pathological sections of IMER group showed no obvious inflammatory cell infiltration in the vital organs and implant-position skin tissue (Figure S32c). Therefore, the encapsulated IMER had no significant pathological changes and toxicity to visceral tissues.

33. Noise level of the IMER

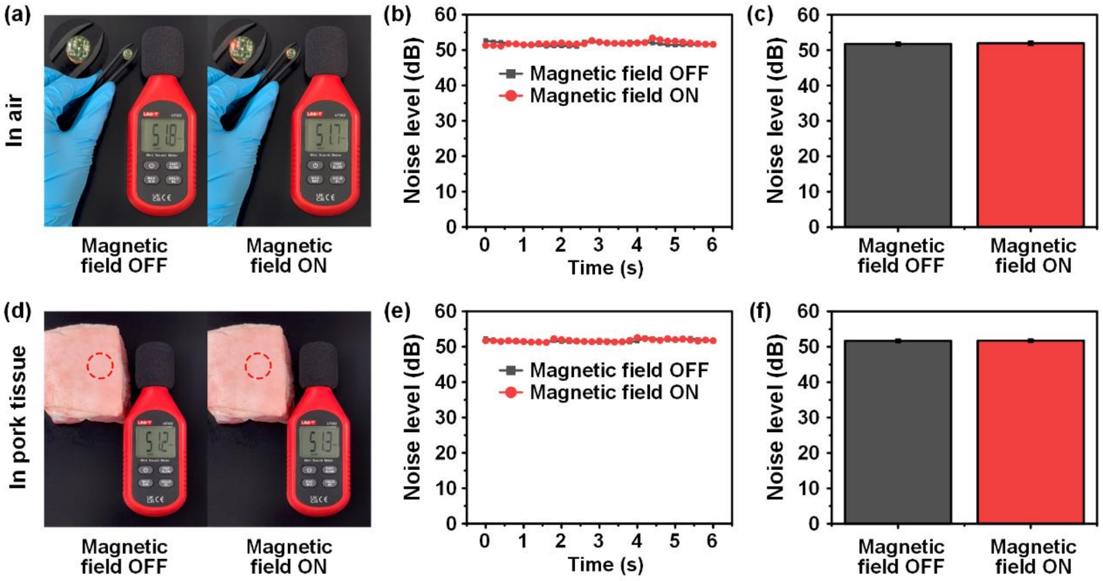

Figure S33. (a) The IMER placed in air under magnetic OFF and ON states, respectively. **(b)** The recorded noise level of the IMER placed in air during 6 s. **(c)** The noise level of the IMER in air in magnetic field OFF and ON states. **(d)** The IMER placed in pork tissue under magnetic OFF and ON states, respectively. **(e)** The recorded noise level of the IMER placed in pork tissue during 6 s. **(f)** The noise level of the IMER in pork tissue in magnetic field OFF and ON states.

To investigate the noise level of the wireless magnetic energy transfer system, the varying sound levels of the rotating IMER were measured by the mini sound meter (UNI-T, UT353), as shown in Figure S32. When the magnetic field was turned on, the magnetic energy transmitted by the IMER drove the LED to emit light (Figure S33a). The noise levels were measured in the magnetic field OFF and ON states, respectively. The noise of the IMER placed in air was recorded continuously for 6 s in Figure S33b and Figure S33c. When the magnetic field was turned OFF, the measured noise fluctuated in the range of 51-52.7 dB. When the magnetic field was turned ON, the measured noise fluctuated in the range of 51.1-53.4 dB. The results showed no significant difference between the two magnetic field states. Additionally, the IMER was placed in the pork tissue, and the noise levels were measured as shown in Figure S33d. The results in Figure S32e and Figure S32f showed that the measured noise fluctuated in the range of 51.2-52.6 dB without difference between the two magnetic field states.

34. Experimental animals and ethics statement

Male Sprague-Dawley rats (8-10 weeks old, 300-400 g, n=8) housed with ad libitum access to food and water under 12-h light/dark cycles were used for experiments. All animal procedures were performed according to the ethical standards approved by the Institutional Animal Care and Use Committee at Sun Yat-Sen University (Approval Number: SYSU-IACUC-2021-000401).

Supplementary Videos

Video S1: The magnetic energy transmission process to power the IMER for LED illumination.

Video S2: Wireless bioelectronic stimulation for sciatic nerve controlled by smartphone APP.

Video S3: Recorded trajectories of the optogenetic rats during optoelectronic stimulation.

Table S1. The structure design and electricity output performance of IMER.

IMER	MIFC	Copper coil					IMER				
	Diameter	Inner diameter	Outer diameter	Height	Wire diameter	Resistance	Volume	Output voltage	Output current	Rectified voltage	Max power
Φ 6×3 mm^3	3 mm	3 mm	6 mm	3.1 mm	0.06 mm	74.5~77.5 Ω	≈ 88 mm^3	0.7~1 V	9~10 mA	1.8~2.2 V	2.5 mW
Φ 8×4 mm^3	4 mm	4 mm	8 mm	4.1 mm	0.06 mm	172.5~175.5 Ω	≈ 206 mm^3	2.6~3 V	12~15 mA	6~7 V	9.6 mW
Φ 10×5 mm^3	5 mm	5 mm	10 mm	5.1 mm	0.06 mm	283.5~286.5 Ω	≈ 400 mm^3	7~8 V	18~20 mA	15.8~16.2 V	14.8 mW

Table S2. Comparison between different wireless energy transfer methods

**Type**	Conversion mode	Tissue depth	Frequency	Advantages	Limiting factors
Ultrasound	Piezoelectric effect<br>Triboelectric effect	1 cm [35]<br>5.5 cm [36]<br>1 cm [37]	kHz-MHz	High power;<br>Deep penetration	Thermal damage<br>Induced cavity
Light	Photovoltaic effect	0.075 cm [24]<br>0.055 cm [33]<br>0.85 cm [34]	~THz	High power	Low penetration;<br>Thermal damage
Heat	Pyroelectric effect	/	/	/	Low power;<br>Low penetration;<br>Thermal damage
Magnetoelectric material	Magnetostrictive effect<br>Piezoelectric effect	4 cm [40]	~kHz	High biosafety	Low power
Electromagnetic radiation	Electromagnetic effect	3 cm [38]<br>0.6 cm [5]	~GHz	Long distance	High equipment requirement;<br>Rapid attenuation;<br>Low efficiency
Magnetic resonance coupling	Electromagnetic effect	0.19 cm [39]<br>11.5 cm [45]	kHz-MHz	High power	High coupling requirement
Magnetic inductive coupling	Electromagnetic effect	0.1 cm [41]<br>0.4 cm [42]	~Hz-kHz	Low equipment requirement	Short distance
ULFMEF<br>(This job)	Enhanced electromagnetic induction coupling	~20 cm	~50 Hz	Deep penetration;<br>Low attenuation;<br>Low thermal damage	Large rigidity;<br>Low biosafety

References

[5] Zaeimbashi M, Nasrollahpour M, Khalifa A *et al*. Ultra-compact dual-band smart NEMS magnetoelectric antennas for simultaneous wireless energy harvesting and magnetic field sensing. *Nat Commun* 2021; **12**: 3141.

[24] Song K, Han JH, Lim T *et al*. Subdermal Flexible Solar Cell Arrays for Powering Medical Electronic Implants. *Adv Healthc Mater* 2016; **5**: 1572–80.

[33] Lyu SZ, He YL, Tao XL *et al*. Subcutaneous power supply by NIR-II light. *Nat Commun* 2022; **13**: 6596.

[34] Jeong J, Jung J, Jung D *et al*. An implantable optogenetic stimulator wirelessly powered by flexible photovoltaics with near-infrared (NIR) light. *Biosens Bioelectron* 2021; **180**: 113139.

[35] Wan X, Chen P, Xu ZS *et al*. Hybrid-Piezoelectret Based Highly Efficient Ultrasonic Energy Harvester for Implantable Electronics. *Adv Funct Mater* 2022; **32**: 2200589.

[36] Piech DK, Johnson BC, Shen K *et al*. A wireless millimetre-scale implantable neural stimulator with ultrasonically powered bidirectional communication. *Nat Biomed Eng* 2020; **4**: 207–22.

[37] Hinchet R, Yoon H-J, Ryu H *et al*. Transcutaneous ultrasound energy harvesting using capacitive triboelectric technology. *Science* 2019; **365**: 491–4.

[38] Bansal A, Yang FY, Xi T *et al*. In vivo wireless photonic photodynamic therapy. *P Natl Acad Sci USA* 2018; **115**: 1469–74.

[39] Sun B, Bte Rahmat JN, Kim HJ *et al*. Wirelessly Activated Nanotherapeutics for In Vivo Programmable Photodynamic-Chemotherapy of Orthotopic Bladder Cancer. *Adv Sci* 2022; **9**: 2200731.

[40] Chen JC, Kan P, Yu ZH *et al*. A wireless millimetric magnetoelectric implant for the endovascular stimulation of peripheral nerves. *Nat Biomed Eng* 2022; **6**: 706–16.

[41] Lee HE, Park JH, Jang D *et al*. Optogenetic brain neuromodulation by stray magnetic field via flash-enhanced magneto-mechano-triboelectric nanogenerator. *Nano Energy* 2020; **75**: 104951.

[42] Guo QL, Koo J, Xie ZQ *et al*. A Bioresorbable Magnetically Coupled System for Low-Frequency Wireless Power Transfer. *Adv Funct Mater* 2019; **29**: 1905451.

[45] Chen P, Wu P, Wan X *et al*. Ultrasound-driven electrical stimulation of peripheral nerves based on implantable piezoelectric thin film nanogenerators. *Nano Energy* 2021; **86**: 106123.
